# Adolescent Medicine Trials Network for HIV/AIDS Interventions Data Harmonization: Rationale and Development of Guidelines

**DOI:** 10.2196/11207

**Published:** 2018-12-21

**Authors:** Micah McCumber, Demetria Cain, Sara LeGrand, Kenneth H Mayer, Debra A Murphy, Matthew A Psioda, Arlene C Seña, Tyrel J Starks, Michael Hudgens

**Affiliations:** 1 Department of Biostatistics Gillings School of Global Public Health University of North Carolina at Chapel Hill Chapel Hill, NC United States; 2 Center for HIV Educational Studies & Training Hunter College New York, NY United States; 3 Duke Global Health Institute Duke University Durham, NC United States; 4 The Fenway Institute Fenway Health Boston, MA United States; 5 Department of Psychiatry University of California at Los Angeles Los Angeles, CA United States; 6 Institute for Global Health and Infectious Diseases Department of Medicine University of North Carolina at Chapel Hill Chapel Hill, NC United States; 7 Department of Psychology Hunter College New York, NY United States

**Keywords:** adolescent, Adolescent Medicine Trials Network for HIV/AIDS Interventions, data harmonization, HIV

## Abstract

**Background:**

The Adolescent Medicine Trials Network for HIV/AIDS Interventions (ATN) research program aims to defeat the rising HIV epidemic among adolescents and young adults in the United States.

**Objective:**

This study aims to optimize cross-study analyses and comparisons of standardized measures (variables) collected in the ATN.

**Methods:**

Guidelines were developed for harmonizing measures to be collected across ATN studies.

**Results:**

Eight domains were identified for harmonization—Demographics and Socioeconomic Characteristics, Sexual Behavior and Risk, Substance Use and Abuse, HIV-Positive Cascade, HIV-Negative Cascade, Mental Health, Social Support and Isolation, and Pre-exposure Prophylaxis Cascade.

**Conclusions:**

The collection of selected key measures in a uniform manner across studies facilitates the characterization of participant populations, comparisons between studies, and pooled analysis of data from multiple studies.

## Introduction

The Adolescent Medicine Trials Network for HIV/AIDS Interventions (ATN) research program aims to defeat the rising HIV epidemic among adolescents and young adults in the United States by increasing awareness of the HIV status and access to health care for those diagnosed with HIV. The ATN develops and conducts behavioral, community-based, translational, therapeutic, microbicide, and vaccine trials in HIV-at-risk and HIV-infected youth aged 12-24 years, with a focus on the inclusion of minors. The ATN research is conducted through collaborations within the network and with researchers in other institutions across the United States. The ATN website [1] provides additional information about the network.

The ATN currently includes 3 research program projects (or U19s) and a Coordinating Center with >20 currently active study protocols across the network. Without standardization, data collected across these different studies may be difficult or impossible to combine. In turn, this could potentially hamper efforts to compare data across studies or describe the US adolescent and youth populations choosing to participate in the ATN research.

Therefore, the ATN Analytic Committee (AC) developed guidelines for harmonizing (ie, standardizing) measures (variables) to be collected across ATN studies to optimize cross-study analyses and comparisons. This set of harmonized measures facilitates pooled analysis of data and allows the characterization and comparison of participants across ATN studies conducted among diverse populations in the United States.

## Methods

### Adolescent Medicine Trials Network for HIV/AIDS Interventions Data Harmonization Process

**Figure 1:**
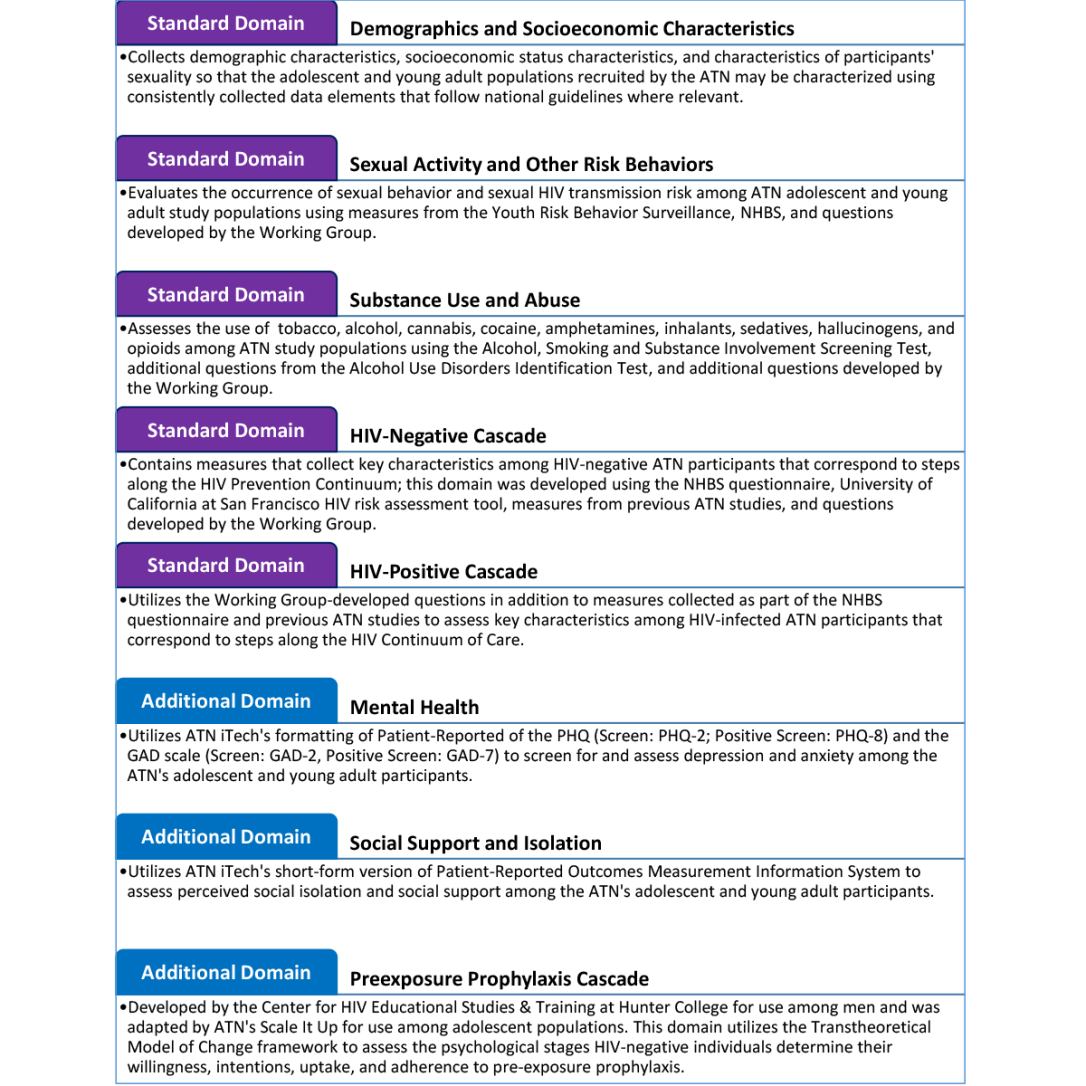
A summary of the domains included in the Adolescent Medicine Trials Network for HIV/AIDS Interventions (ATN) data harmonization guidelines. GAD: Generalized Anxiety Disorder; NHBS: National HIV Behavioral Surveillance System; PHQ: Patient Health Questionnaire.

The AC developed a set of harmonized measures to be collected across the diverse set of projects in the ATN. Eight domains were identified for harmonization: 5 “standard” domains for which characteristics (measures, variables) will be collected in all ATN studies unless a strong operational or scientific rationale exists otherwise; and 3 “additional” domains for studies planning to collect data in these domains (Figure 1). The data harmonization guidelines focus primarily on survey questions and measures and include recommendations for the order in which the measures should be collected, as well as the ordering of levels for particular measures.

The 5 standard domains were developed by reviewing common measures collected by previous and current ATN studies and identifying key areas of interest among ATN studies; these domains included “Demographics and Socioeconomic Characteristics,” “Sexual Behavior and Risk,” “Substance Use and Abuse,” “HIV-Negative Cascade,” and “HIV-Positive Cascade.” The 3 additional measure domains that were identified for harmonization across ATN studies included “Mental Health,” “Social Support and Isolation,” and the “Motivational Pre-exposure Prophylaxis (PrEP) Cascade.” These additional measures are not required but recommended for studies that plan to collect related information. The ATN data harmonization guidelines were reviewed and received the final approval by the ATN Executive Committee (EC). The following paragraphs describe the process undertaken by the ATN AC for identifying and selecting measures to include within each domain before submission to the ATN EC for approval.

### Standard Domains

The “Demographics and Socioeconomic Characteristics” domain was developed by the collective AC by compiling common survey questions and measures collected in current and previous ATN studies. A draft of the demographic and socioeconomic measures was then distributed to AC members for discussion. The AC members provided their feedback and measures were discussed during biweekly calls. The AC removed measures from the standard domains that could not obtain AC and EC consensus, but these measures remain available for optional use by future ATN studies.

The AC identified “Substance Use and Abuse,” “Sexual Behavior and Risk,” “HIV-Negative Cascade,” and “HIV-Positive Cascade” as critical domains to be developed by smaller working groups. To facilitate the creation of working groups for each of these 4 domains, U19 team members indicated their willingness to participate in or lead any of the 4 working groups. These working groups were charged with 3 objectives as follows: (1) review currently planned and previously conducted ATN studies to gain a broad understanding of data points within the domain that might be collected in future ATN research studies; (2) evaluate potential data items for the utility and feasibility of collection in a standardized manner in upcoming ATN research studies; and (3) recommend a core set of data items for collection in upcoming ATN studies and provide additional recommendations that might be used in some but not all studies, if appropriate. The working groups held calls as needed to review the existing measures and literature and develop a list of proposed questions and measures to include in their assigned domain. The recommended questions and measures were then presented to the larger AC for feedback and approval.

The Sexual Activity and Other Risk Behaviors Working Group sought to determine a minimum number of data points with broad relevance by utilizing data measures from the Youth Risk Behavior Surveillance (YRBS) [2,3].

Prior to the formation of the working groups, the AC agreed that using the Alcohol, Smoking and Substance Involvement Screening Test (ASSIST) would be a good starting place for the “Substance Use and Abuse” domain [4]; this decision was largely based on the use of the ASSIST instrument in previous ATN studies. The Substance Use and Abuse Working Group then identified additional variables that are often included in ATN studies’ data collection instruments but are not already collected in the ASSIST.

The “HIV-Negative Cascade” and “HIV-Positive Cascade” domains are critical components to the data harmonization guidelines because the ATN research agenda is focused on the prevention and treatment care continua. Regarding the HIV prevention cascade, the ATN seeks to develop and examine the feasibility and potential impact of the delivery of novel services, delivery of services in novel settings, and the use of novel engagement strategies for reaching high-risk youth and promoting the uptake of essential services such as HIV testing, sexually transmitted infections (STI) testing, risk screening, condom distribution, PrEP, and postexposure prophylaxis (PEP).

For the HIV treatment and care cascade, the ATN seeks to determine the most effective strategy or set of strategies for linking positive youth to care, promoting retention in care (including antiretroviral uptake and adherence), and obtaining and sustaining viral suppression. These research goals motivated the selection of many of the measures included in the “HIV-Negative Cascade” and “HIV-Positive Cascade” domains, as well as in other standard domains. The HIV-Negative Cascade and HIV-Positive Cascade Working Groups used measures from existing surveys, like the National HIV Behavioral Surveillance (NHBS) survey [5], and some additional customized data items used in previous ATN studies to define a minimum set of harmonized measures that would capture information along the continua.

### Additional Domains

For the 3 additional domains for harmonization (Mental Health, Social Support and Isolation, and Motivational PrEP Cascade), the process was streamlined. U19 representatives proposed sets of measures that were already harmonized within their U19. The Patient Health Questionnaire and the Generalized Anxiety Disorder Scale (GAD) were proposed to measure mental health [6-10], while the Patient-Reported Outcomes Measurement Information System (PROMIS) measures were proposed to measure social support and social isolation [11]. The Motivational PrEP Cascade measures were adapted for use with adolescents from measures developed by the Center for HIV Educational Studies & Training at Hunter College for use in HIV-negative men who have sex with men (MSM) [12]. The AC reviewed and approved the compiled measures before receiving the final approval from the ATN EC.

## Results

### Adolescent Medicine Trials Network for HIV/AIDS Interventions Data Harmonization Guidelines

The ATN data harmonization guidelines describe general guidelines in addition to domain-specific harmonizing measures or variables to be collected across ongoing and upcoming ATN studies (Multimedia Appendix 1). These guidelines provide specific formatting and skip pattern information for standardization across the ATN. Standardized data fields and datasets are critical to enabling comparisons across studies and analyses that combine data from multiple network studies.

The general data harmonization guidelines are as follows:

1. For the 5 standard domains, all characteristics (measures, variables) should be collected in all ATN studies, except when there is a strong operational or scientific rationale to exclude them. Measures for the 3 additional domains (Mental Health, Social Support and Isolation, and the Motivational PrEP Cascade) are recommended for studies planning to collect data in these domains.
2. Conventions for nonresponse will be handled on a per study basis. Thus, where relevant, the set of acceptable values (levels or responses) for a given variable may be augmented with additional values such as “Don’t know” or “Unsure.” Individual study teams are encouraged to develop data collection strategies that minimize nonresponse or missing data.
3. In general, the order of characteristics does not reflect any suggested ordering for data collection, unless indicated otherwise. For example, the current National Institute of Health (NIH) and Food and Drug Administration guideline recommends a 2-question format for requesting race and ethnicity information, with the ethnicity question preceding the question about race.
4. For a particular characteristic, the following is recommended regarding the ordering of the levels (possible responses). First, following Clinical Data Acquisition Standards Harmonization recommendations, a consistent order of responses should be used from question to question; exceptions to this would be cases where a validated instrument (eg, a standardized assessment questionnaire) is used. If there is a logical sequential order (ie, ordinal variables), as in the current educational level, order the levels accordingly. For nonordinal variables, order according to the anticipated likelihood of response level or alphabetically.
5. Possible response values can be separated further, if finer details are desired. However, more detailed possible response values should be designed in a way that they can be aggregated to match the values listed for each of the harmonized data items in Multimedia Appendix 1.
6. Skip patterns are highlighted in blue within the data harmonization tables provided in Multimedia Appendix 1.

### Standard Domains

#### Demographic and Socioeconomic Characteristics

The initial data domain selected for ATN-wide harmonization included demographic characteristics, socioeconomic status characteristics, and characteristics of participant sexuality (Table 1 in Multimedia Appendix 1). These characteristics were identified to be of primary importance owing to their relevance to virtually all ATN studies and the desire of the ATN to be able to characterize the adolescent populations recruited by the ATN using consistently collected data elements. Race and ethnicity data were collected in accordance with the current Food and Drug Administration guidelines and NIH policy [13,14]; in accordance with that policy, ethnicity data were solicited first, and the race was collected in a check-all-that-apply format. The ZIP code for the location at which each participant primarily lives was collected to allow linkage to census track data. In addition, data on each participant’s current gender identity, sex assigned at birth, and sexual identity were collected. The possible responses to these questions were designed to be consistent with the National Coalition for Sexual Health guidelines for health care providers and customized for adolescent populations served by the ATN [15]. Owing to the significant footprint of the ATN in the Lesbian, Gay, Bisexual, Transgender, Queer adolescent community, we collected several data elements regarding the degree to which immediate family members and peers are aware of participants’ sexual identity. A supportive family environment has been shown to be highly influential regarding a Lesbian, Gay, Bisexual, Transgender, Queer adolescent’s mental and physical health and risk-taking behavior [16-18]. Furthermore, data elements related to whether a participant is currently in school or working, their level of education, and their health insurance coverage were collected to characterize the socioeconomic status.

#### Sexual Behavior and Risk

The Sexual Behavior and Risk domain aims to evaluate the occurrence of sexual behavior and sexual HIV transmission risk among the general ATN study populations. The content selection was informed by data from the YRBS, which identified low rates of condom use, a high number of sexual partners, and concurrent substance use during sex among the challenges to HIV prevention among youth [2]. To maximize comparability to existing national datasets on sexual behavior among youth, the working group included members who were familiar with the implementation of sexual behavior measures utilized in the YRBS. Wherever possible, ATN-harmonized questions were designed to be comparable to YRBS. It is recommended that the characteristics listed be collected in the order presented in Table 2 of Multimedia Appendix 1.

To reduce participant demand, the working group opted to collect count-data on the number of sexual partners (lifetime and past 3 months) but not on the number of sexual events. Because questions on the biomedical prevention uptake were developed as part of the HIV-Negative Cascade, this domain focused on the assessment of condom use during sex. Again, to minimize the participants’ burden, the decision was made to prioritize identifying the mere occurrence of recent (past 3 month) condomless sex rather than quantifying the amount of risk (eg, the number of condomless sex events). To do this, a series of “yes“ or ”no” questions asking about condomless sex with partners whose HIV status was known (either HIV-negative or HIV-positive) or unknown were included in the harmonized measures. In lieu of collecting data on the number of condomless sex acts, a visual analog scale was utilized to query the percentage of time participants utilized condoms while having sex in the past 3 months. Finally, the working group incorporated a single item inquiring about the lifetime occurrence of either alcohol or drug use during vaginal or anal sex.

The working group acknowledges that the harmonized sexual behavior and risk measures are limited in their nature. Studies within the ATN vary in their emphasis on sex; thus, nuanced data on sexual behavior would represent an unnecessary burden on participants in some studies. In contrast, studies aimed at achieving reductions in the sexual HIV transmission risk may need substantially more detail in their data collection. For these studies, the working group recommended the AIDS Behavior Risk Assessment, the YRBS, or the NHBS questionnaire as additional resources [2,5,19-21].

#### Substance Use and Abuse

Many adolescents experiment with the use of alcohol or other illicit substances [22,23]. The use of such substances has been shown to be associated with an increase in risky sexual behavior and, therefore, a greater risk for HIV transmission [24-26]. The ATN adapted several standardized instruments to assess alcohol and nonprescription drug use for use in studies of adolescents (Table 3 in Multimedia Appendix 1). The ATN data collection instrument was primarily derived from the ASSIST [4,27,28], which has been used in previous ATN studies (eg, ATN 071). As its name suggests, the ASSIST is a screening tool for substance use to be used in a clinical setting. For the ATN, it was adapted for computer-assisted self-interview as a means of quantifying the degree of substance use for adolescent participants and to collect basic information on the impact of substance abuse on their daily lives. In ATN studies where site staff will administer the ASSIST, the Substance Use and Abuse Working Group recommended using the traditional, interviewer-administered ASSIST format, which is designed for that type of implementation [23].

Substance use was assessed for the following classes of substances: tobacco, alcohol, cannabis, cocaine, amphetamines, inhalants, sedatives, hallucinogens, and opioids. For most substances, ≥5 examples were given using terminology appropriate for the adolescent populations studied by the ATN (eg, Vicodin instead of acetaminophen-hydrocodone). Explanatory prompts were added owing to the self-interview format required for most ATN studies. The data collection on opioid use was augmented by the addition of 2 questions regarding the specific use of heroin during the current epidemic in the United States [29]. The data collection on alcohol use was augmented by the addition of 2 questions derived from the Alcohol Use Disorders Identification Test (AUDIT-C) to assess binge drinking owing to its prevalence in adolescent populations [29,30]. The definition of binge drinking used for the data collection was taken from the National Institute of Alcohol Abuse and Alcoholism [31].

For each class of substance, the harmonized measures assess the lifetime use (item 1) and aspects of usage over the past 3 months (items 2-5). Per the recommendation of the Substance Use and Abuse Working Group, ATN studies can also collect data on the aspects of usage using a frame of reference of the past 12 months to better align with the NHBS study, if desired. Substance use data are collected by the ATN for 2 purposes as follows: (1) to quantify the prevalence of substance in the populations studied; and (2) to facilitate risk adjustment in analyses that are more central to ATN research aims. The ASSIST provides an appealing solution for the second purpose. Each question on the ASSIST has a set of responses to choose from, and each response from questions 2-7 has a numerical score. The scores from questions 2-7 are added across each substance (eg, tobacco, alcohol, or cannabis) to produce an ASSIST risk score for each substance. In technical reports and papers, this score was referred to as the specific substance involvement score for each drug class. More details on scoring can be found in the ASSIST manual [4]. The translation of individual survey responses to a summary risk score is appealing for the ATN as this provides a standardized framework for performing risk adjustment for relevant analyses in ATN studies. In contrast, survey items from large national surveys, such as Monitoring the Future [22,23,32], assess substance use for the primary purpose of characterizing the prevalence of use in US adolescents and, therefore, may be less useful for research aims that are of primary importance to the ATN.

#### HIV-Negative Cascade

The HIV-Negative Cascade focuses on the prevention of HIV among adolescents and includes measures related to HIV testing, STI testing, PEP and PrEP awareness, utilization, adherence, and barriers to PrEP utilization and adherence (Table 4 in Multimedia Appendix 1). General HIV testing and STI questions for the HIV-Negative Cascade were taken from the NHBS questionnaire. The Centers for Disease Control and Prevention (CDC), in collaboration with 25 state and local health departments, began the NHBS in 2003. The NHBS was designed to conduct behavioral surveillance among persons at high risk for HIV infection and surveyed the 3 populations at highest risk for HIV in the United States—MSM, intravenous drug use, and high-risk heterosexuals [20,33]. PEP and PrEP awareness and utilization questions were taken from the University of California at San Francisco HIV risk assessment tool, NHBS [5], and assessments used in the ATN’s Scale It Up [12] and CARES studies.

For studies planning to collect more detailed information related to PrEP, the AC recommended harmonized measures from the Motivational PrEP Cascade as additional measures (Table 9 in Multimedia Appendix 1). The NHBS instrument might also be considered for studies that will collect measures related to the HIV-Negative Cascade at a more detailed level than the required harmonized measures [5]. This questionnaire makes extensive use of skip patterns to ask questions specific to gender identity and sexual orientation. The NHBS data provide behavioral context trends in HIV surveillance data and describe populations in the United States at increased risk for HIV infection.

#### HIV-Positive Cascade

This domain was developed with the goal of identifying key characteristics among HIV-infected participants that correspond to steps along the HIV Continuum of Care, using common definitions developed by the Health Resources and Services Administration HIV/AIDS Bureau and the CDC. The steps in the continuum are typically defined using biomedical data collected during clinical care for HIV-infected patients, including the CD4 count and HIV viral load before and after initiation of antiretroviral therapy (ART). Linkage to care, for example, is defined as having ≥1 documented CD4 or viral load measures within 30 days (1 month) of diagnosis, while retention is defined as having ≥2 viral load or CD4 tests in the last year, performed, at least, 3 months apart.

The participant questions in this domain primarily address medical appointments with HIV providers, missed visits, CD4 and viral load testing, and adherence to ART, using questions derived from several sources, including the NHBS and prior ATN studies (Tables 5 and 6 in Multimedia Appendix 1). In addition, the HIV-Positive Cascade Working Group developed 4 additional questions for data harmonization that capture participants’ characteristics corresponding to reengagement and retention in care based on missed appointments over time.

Furthermore, the NHBS survey instrument might be considered for studies that will collect measures related to the HIV-Positive Cascade at a more detailed level than the required harmonized measures [5]. For studies collecting CD4 count from biomedical data, it is recommended that investigators collect the CD4 collection date, CD4^+^ T-cell absolute count (cells/mm^3^), CD4‑ T-cell percent (%), and data source (similar to Viral Load Data Source; Table 6 in Multimedia Appendix 1).

### Additional Domains

#### Mental Health

In the United States, anxiety and depression are among the most common mental health disorders for adolescents and young adults [34]. For HIV-positive youth, anxiety and depression have been associated with poorer medication adherence and decreased viral suppression [35-40]. Furthermore, direct and indirect relationships between depression and anxiety and increased sexual risk behaviors among youth have been identified in several studies [41], though other studies have found null or conflicting findings.

The Mental Health domain collects data on anxiety and depression using a 2-step approach. All study participants complete the 2-item Patient Hospital Questionnaire (PHQ-2) and the 2-item Generalized Anxiety Disorder Scale (GAD-2), which are used to screen for depression and anxiety, respectively. The PHQ-2 consists of the first 2 items of the 8-item version of the questionnaire, the PHQ-8, and assesses the 2 core criteria for depressive disorders. The PHQ-2 has good operating characteristics (eg, sensitivity and specificity) for detecting depressive disorders [6,7]. The GAD-2 includes the first 2 items of the 7-item version of the scale, the GAD-7, and assesses the 2 core criteria for generalized anxiety disorder. In addition, the GAD-2 items have been found to be appropriate screening items for panic, social anxiety, and posttraumatic stress disorders. The GAD-2 has good operating characteristics for screening for all 4 types of anxiety disorders [7,8]. For study participants who screen positive on the PHQ-2 or GAD-2, the remaining items of the PHQ-8 or GAD-7 are administered to determine the symptom severity (described in Table 7 of Multimedia Appendix 1). Furthermore, the longer PHQ-8 and GAD-7 with broader scoring ranges may be useful for examining changes in depression and anxiety over time. Both the PHQ-8 and the GAD-7 have been shown to be reliable and valid measures [7-10].

#### Social Support and Isolation

The Social Support and Isolation domain uses the PROMIS short-form versions of the Social Relationships scales to measure perceived social isolation and social support [42]. PROMIS, an NIH initiative, uses rigorous processes to develop and test item banks that measure physical, mental, and social health components [11]. The 5 Social Relationships short-form scales, each with 4 items, measure domains of social isolation and social support, including companionship, emotional support, informational support, and instrumental support [42]. The Social Support and Isolation measures are included in Table 8 of Multimedia Appendix 1.

#### Motivational Pre-exposure Prophylaxis Cascade

The Motivational PrEP Cascade domain and measurements utilize the Transtheoretical Model of Change framework to assess the psychological stages HIV-negative individuals determine their willingness, intentions, uptake, and adherence to PrEP [43]. The development of the Motivational PrEP Cascade was based on the formative work suggesting that, among individuals *willing* to take PrEP (ie, those for whom PrEP acceptability is high), there was wide variability in behavioral *intentions* to do so [44]. The Motivational PrEP Cascade complements the HIV-Negative and HIV-Positive Cascades with the overall goal of identifying facilitators and barriers to the PrEP uptake needed for addressing implementation issues. The measurements have been tested on a national sample of HIV-negative gay and bisexual men in the United States with results identifying fewer than 1 in 10 as currently using and adhering to PrEP [12]. Based on this initial work, questions were adapted for youth and a reduced 15-item version of the scale containing only questions considered essential to estimating progress along the PrEP Cascade was selected for inclusion in the harmonized ATN measures (Table 9 in Multimedia Appendix 1).

There are 5 stages to the Motivational PrEP Cascade that reflect decision-making processes across time. The cascade is most appropriate for samples of objectively identified PrEP candidates based on the risk for HIV infection using the established CDC criteria [45]. The inclusion of individuals into the cascade who are not at risk for HIV infection may confound accurate prevention numbers. Stage 1 is PrEP precontemplation and includes individuals who are objectively identified as PrEP candidates, but do not view themselves as good candidates for PrEP or are unwilling to pursue PrEP. Those who do not meet the criteria for stage 2 are considered PrEP precontemplation. Stage 2: PrEP contemplation includes those who identify themselves as PrEP candidates (Table 9: PrEP_Q1) and willing to pursue PrEP. Willingness was defined as those indicating they would probably or definitely take PrEP if they could get it for free and without their parent’s knowing (Table 9: PrEP_Q6 and PrEP_Q7). Stage 3: Preparation includes those who intend to start PrEP but have not yet started (Table 9: PrEP_Q8) and who know of a medical provider that would prescribe PrEP (Table 9: PrEP_Q10). Those indicating they would definitely or probably start taking PrEP were coded as intending to begin PrEP. Those who had talked to a medical provider about starting PrEP and both thought they should start PrEP (Table 9: PrEP_Q11) and those who are currently on PrEP (Table 9: PrEP_Q2) are in Stage 4: PrEP Action. Stage 5: PrEP Maintenance includes those who are currently prescribed PrEP (Table 9: PrEP_Q2), adherent to their regimen (Table 9: PrEP_Q14), and receiving quarterly HIV or STI testing (Table 9: PrEP_Q15).

## Discussion

The ATN developed guidelines for harmonizing standard measures for Demographics and Socioeconomic Characteristics, Sexual Behavior and Risk, Substance Use and Abuse, HIV-Negative Cascade, and HIV-Positive Cascade domains. In addition, guidelines for additional measures commonly collected among ATN studies were developed for Mental Health, Social Support and Isolation, and PrEP Cascade domains. The research goals of the ATN motivated many of the measures included in these standard domains, especially the measures for the HIV-Negative Cascade and HIV-Positive Cascade domains. AC and working group members referred to existing surveys and data collection tools, like YRBS, ASSIST, NHBS and PROMIS, to develop the ATN harmonization guidelines. As the ATN works to increase awareness of the HIV status and access to health care for adolescents diagnosed with HIV in the United States, the collection of selected key measures uniformly across studies facilitates the characterization of participant populations, comparisons between studies, and pooled analysis of data from multiple studies.

Moving forward, the ATN should periodically evaluate the utility of each of the harmonized measures currently being collected across ATN studies and update these data harmonization guidelines as needed. Some of the measures currently included are relatively new and do not necessarily have a robust evidence base supporting their validity and reliability, especially in adolescents and young adults in the United States. Therefore, it will be important for the ATN to reassess these harmonized measures in the future. In addition, gaps may be identified that warrant the inclusion of further measures. For example, the current harmonized measures do not consider stigma or cost-effectiveness. Related to cost-effectiveness, the ATN Modeling Core recently formed a working group to explore the feasibility of standardizing cost-related measures collected in ATN studies or developing guidelines for harmonizing cost-effectiveness analyses across the network. Like the continuously evolving HIV epidemic, the ATN-harmonized measures should evolve as well to ensure the collection of data most relevant to defeating the HIV epidemic among adolescents and young adults.

## Appendix

Multimedia Appendix 1 The measures (variables) defined as part of the Adolescent Medicine Trials Network for HIV/AIDS Interventions (ATN) Data Harmonization Guidelines.

## Abbreviations

AC: Analytic Committee
ASSIST: Alcohol, Smoking and Substance Involvement Screening Test
ATN: Adolescent Medicine Trials Network for HIV/AIDS Interventions
CDC: Centers for Disease Control and Prevention
EC: Executive Committee
GAD-2: Two-item Generalized Anxiety Disorder Scale
GAD-7: Seven-item Generalized Anxiety Disorder Scale
NHBS: National HIV Behavioral Surveillance System
NIH: National Institute of Health
PEP: postexposure prophylaxis
PHQ-2: Two-item Patient Hospital Questionnaire
PHQ-8: Eight-item Patient Hospital Questionnaire
PrEP: Pre-exposure prophylaxis
PROMIS: Patient-Reported Outcomes Measurement Information System
STI: sexually transmitted infections
YRBS: Youth Risk Behavior Surveillance
