# Supplementary material for: Adolescent Medicine Trials Network for HIV/AIDS Interventions Data Harmonization: Rationale and Development of Guidelines
Source: JMIR Res Protoc. 2018 Dec 21;7(12):e11207. doi: 10.2196/11207 (PMC6320398; doi:10.2196/11207)
Supplement: Multimedia Appendix 1 [file resprot_v7i12e11207_app1.pdf]

Table 1: ATN Standard Domain: Demographic and Socioeconomic Characteristics

Demographic and Socioeconomic Characteristics		
**Characteristic**	**Format/Values**	**Comments**
**Birth Date**<br>What is your date of birth? (enter month, day, year)	MMDDYYYY	
**Ethnicity**<br>Do you consider yourself Hispanic or Latino?	Coded Field:<br>1. Yes<br>2. No	The ethnicity question should precede the question about race. The ordering and format recommended here is consistent with FDA guidance (issued Oct 26, 2016)[1] on the collection of race and ethnicity in clinical trials and with NIH guidance[2].
*Asked if **Ethnicity** = **Yes***<br><br>**Ethnic Ancestry**<br>What best describes your Hispanic or Latino ancestry? (*One or more categories may be selected*)	Coded Field:<br>1. Cuban<br>2. Dominican<br>3. Mexican<br>4. Puerto Rican<br>5. Other, specify: ______________	
**Race**<br>What is your race? (*Select all that apply*)	Coded Field:<br>1. American Indian or Alaska Native<br>2. Asian<br>3. Black or African American<br>4. Native Hawaiian or Other Pacific Islander<br>5. White<br>6. Other, specify: ______________	
**ZIP Code**<br>What is the 5-digit ZIP code for the location where you primarily live?	Integer	
**Gender Identity**<br>What is your current gender identity?	Coded Field:<br>1. Female<br>2. Male<br>3. Trans female or trans woman<br>4. Trans male or trans man<br>5. Genderqueer or gender nonconforming<br>6. Other, specify: ______________	These categories are consistent with the National Coalition for Sexual Health guideline on the "Sexual Health and Your Patients: A Provider's Guide"[3]
**Assigned Sex at Birth**<br>What sex were you assigned at birth, on your original birth certificate?	Coded Field:<br>1. Female<br>2. Male	

1 http://www.fda.gov/ucm/groups/fdagov-public/@fdagov-afda-gen/documents/document/ucm126396.pdf

2 http://grants.nih.gov/grants/funding/women_min/guidelines_amended_10_2001.htm

3 http://www.ncshguide.org/providers

Demographic and Socioeconomic Characteristics		
**Characteristic**	**Format/Values**	**Comments**
**Sexual Identity**<br>What is your current sexual identity?<br>(*Select all that apply*)	Coded Field:<br>1. Bisexual<br>2. Gay, queer, same gender loving, or homosexual<br>3. Straight or heterosexual<br>4. Other, specify: ______________	Possible response values can be separated, if finer details are desired.<br>For example:<br>1. Bisexual<br>2. Gay<br>3. Homosexual<br>4. Queer<br>5. Same gender loving,<br>6. Straight or heterosexual<br>7. Other, specify: _________
**Family Awareness of Sexual Identity**<br>How many of your immediate family members know your sexual identity?<br><br>*"Immediate family members" includes family members you live with or family members you interact with often.*<br><br>*If you do not have regular contact with any family members, please select "Does not apply".*<br><br>An optional skip pattern may be implemented to only ask this question to participants who indicate their **Sexual Identity** is not **Straight or heterosexual**.	Coded Field:<br>1. None<br>2. Some, but less than half<br>3. About half<br>4. More than half<br>5. All<br>6. Does not apply	
**Peer Awareness of Sexual Identity**<br>How many of your peers know your sexual identity?<br><br>*"Peers" includes your friends, co-workers, and schoolmates.*<br><br>An optional skip pattern may be implemented to only ask this question to participants who indicate their **Sexual Identity** is not **Straight or heterosexual**.	Coded Field:<br>1. None<br>2. Some, but less than half<br>3. About half<br>4. More than half<br>5. All	
**School Enrollment Status**<br>Are you enrolled in school?<br><br>*"School" could mean a school or program where you are working toward a high school diploma, GED, or college/technical degree.*	Coded Field:<br>1. Yes<br>2. No	

Demographic and Socioeconomic Characteristics		
**Characteristic**	**Format/Values**	**Comments**
Asked if **School Enrollment Status = Yes**<br><br>**Current Education Level**<br>What is your current grade level in school?	Coded Field:<br>1. 6th-8th grade<br>2. 9th-12th grade<br>3. GED program<br>4. Technical or vocational school<br>5. Two-year college<br>6. Four-year college<br>7. Graduate school	
Asked if **School Enrollment Status = No**<br><br>**Highest Education Level Completed**<br>What is the highest level of school that you have completed?	Coded Field:<br>1. None, no formal schooling<br>2. 6th-8th grade<br>3. 9th-11th grade<br>4. High school diploma<br>5. High school certificate of completion (no diploma)<br>6. GED<br>7. Some college, technical school, or vocational school<br>8. Technical or vocational school graduate<br>9. Two-year college graduate<br>10. Four-year college graduate<br>11. Some graduate school<br>12. Master's degree or above	
**Current Employment**<br>Are you currently employed?	Coded Field:<br>1. Yes<br>2. No	
Asked if **Current Employment = Yes**<br><br>**Current Employment Type**<br>Are you employed full-time or part-time?<br><br>*Part-time means that you work less than 35 hours per week during most weeks.*	Coded Field:<br>1. Full-time<br>2. Part-time	
**Health Insurance Status**<br>Do you currently have health insurance or health care coverage?<br><br>*This includes private health insurance (e.g., Blue Cross Blue Shield, parent's private insurance) and public health care insurance or coverage (e.g., Medicaid, Medicare).*	Coded Field:<br>1. Yes, I have my own<br>2. Yes, I am covered by my parent/guardian<br>3. No<br>4. Don't Know	

Table 2: ATN Standard Domain: Sexual Behavior and Risk

**Sexual Behavior and Risk**		
**Characteristic**	**Format/Values**	**Comments**
Have you ever had sexual intercourse with somebody else?<br><br>*By sexual intercourse, we mean vaginal or anal sex. By vaginal sex, we mean when one person's penis is inside their partner's vagina. By anal sex, we mean when one person's penis is inside their partner's anus or rectum.*<br><br>*If 2. No, then END Sexual Behavior and Risk questions.*	Coded Field:<br>1. Yes<br>2. No	This question might be used at the screening visit for studies which require previous sexual intercourse as an inclusion criteria.
How old were you when you had sexual intercourse for the first time with somebody else?	Integer	Include logic check that response does not exceed current age
During your life, who have you had sexual intercourse with?	Coded Field:<br>1. Females<br>2. Males<br>3. Females and males	
Ask if sexual intercourse with females or females and males<br><br>During your life, how many **females** have you had sexual intercourse with?	Integer	
Ask if sexual intercourse with males or females and males<br><br>During your life, how many **males** have you had sexual intercourse with?	Integer	
Ask if sexual intercourse with females or females and males<br><br>During the past 3 months, how many **females** did you have sexual intercourse with?	Integer	Logic check should be included to ensure response does not exceed response to "During your life, how many **females** have you had sexual intercourse with?"
Ask if sexual intercourse with males or females and males<br><br>During the past 3 months, how many **males** did you have sexual intercourse with?	Integer	Logic check should be included to ensure response does not exceed response to "During your life, how many **males** have you had sexual intercourse with?"
Did you drink alcohol or use drugs before you had sexual intercourse **the last time**?	Coded Field:<br>1. Yes<br>2. No	

Sexual Behavior and Risk		
**Characteristic**	**Format/Values**	**Comments**
**No Condom Lifetime**<br>In your life, have you ever had vaginal or anal sex <u>WITHOUT</u> using a condom?	Coded Field:<br>**1.** Yes<br>**2.** No	**Source:** NHBS adapted to lifetime question
*Ask if **No Condom Lifetime** is **Yes***<br><br>**No Condom HIV Negative**<br>In the past 3 months, did you have vaginal or anal sex <u>WITHOUT</u> a condom with a partner you knew was HIV negative?	Coded Field:<br>**1.** Yes<br>**2.** No	**Source:** NHBS – changed time period to 3 months
*Ask if **No Condom Lifetime** is **Yes***<br><br>**No Condom HIV Positive**<br>In the past 3 months, did you have vaginal or anal sex <u>WITHOUT</u> a condom with a partner you knew was HIV positive?	Coded Field:<br>1. Yes<br>2. No	**Source:** NHBS – changed time period to 3 months
*Ask if **No Condom Lifetime** is **Yes***<br><br>**No Condom HIV Unknown**<br>In the past 3 months, did you have vaginal or anal sex <u>WITHOUT</u> a condom with a partner whose HIV status you didn't know?	Coded Field:<br>1. Yes<br>2. No	**Source:** NHBS – changed time period to 3 months
**Condom Use Frequency**<br>While looking at the scale below, what percent of time did you use a condom when having vaginal or anal sex in the past 3 months?<br><br>*0% would mean 'NONE' of the time and 100% would mean 'ALL' of the time. If you are unsure, make a best guess.*<br><br>Visual Analog Scale:<br>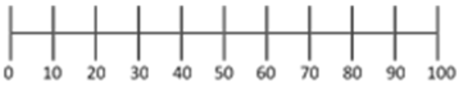	Coded Field:<br>1. 0%<br>2. 10%<br>3. 20%<br>4. 30%<br>5. 40%<br>6. 50%<br>7. 60%<br>8. 70%<br>9. 80%<br>10. 90%<br>11. 100%	**Source:** Changed NHBS frequency of condom use to VAS format

Table 3: ATN Standard Domain: Substance Use and Abuse

Substance Use and Abuse		
**Characteristic**	**Format/Values**	**Source/ Comments**
**Introductory Script:**<br>The following questions are about your experiences using alcohol, tobacco products, and other drugs. Please be assured that this information will be treated as *strictly confidential*.<br><br>The questions will concern your experiences with using these substances across your lifetime and in the past three months. These substances can be smoked, swallowed, snorted, inhaled, injected, or taken in the form of pills. Some of the substances may be prescribed by a doctor (like amphetamines, sedatives, or pain medications). For this survey, do not report on medications that are used as prescribed by your doctor. However, if you have taken such medications for reasons other than prescription, or taken them more frequently or at higher doses than prescribed, please report on this use. We will also ask about cannabis (marijuana) or synthetic cannabis use. If you use or have used these drugs, please report on this use even if you have a prescription or cannabis use is legal in your state or area.<br><br>Once again, please remember that all information provided will be treated as *strictly confidential*.		
**TOBACCO_1: Lifetime use**<br>In your life, have you ever used tobacco products (cigarettes, chewing tobacco, cigars, e-cigarettes, etc.)?<br><br>If **TOBACCO_1=No** then skip to **ALCOHOL_1**.	Coded Fields:<br>1: Yes<br>2: No	ATN 071 / Assist
**TOBACCO_2: Use in past 3 months**<br>In the past 3 months, how often have you used tobacco products (cigarettes, chewing tobacco, cigars, e-cigarettes, etc.)?<br><br>If **TOBACCO_2 = Never** then skip **TOBACCO_3-TOBACCO_5**.	Coded Fields:<br>1: Never<br>2: Once or Twice<br>3. Monthly<br>4: Weekly<br>5: Daily or Almost Daily	ATN 071 / Assist
**TOBACCO_3: Desire to use in past 3 months**<br>During the past 3 months, how often have you had a desire to use tobacco products (cigarettes, chewing tobacco, cigars, e-cigarettes, etc.)?	Coded Fields:<br>1: Never<br>2: Once or Twice<br>3. Monthly<br>4: Weekly<br>5: Daily or Almost Daily	ATN 071 / Assist
**TOBACCO_4: Problems due to use in past 3 months**<br>During the past 3 months, how often has your use of tobacco products (cigarettes, chewing tobacco, cigars, e-cigarettes, etc.) led to health, social, legal or financial problems?	Coded Fields:<br>1: Never<br>2: Once or Twice<br>3. Monthly<br>4: Weekly<br>5: Daily or Almost Daily	ATN 071 / Assist
**TOBACCO_5: Failed to meet expectations in past 3 months**<br>During the past 3 months, how often have you failed to do what was normally expected of you because of your use of tobacco products (cigarettes, chewing tobacco, cigars, e-cigarettes, etc.)?	Coded Fields:<br>1: Never<br>2: Once or Twice<br>3. Monthly<br>4: Weekly<br>5: Daily or Almost Daily	ATN 071 / Assist
**TOBACCO_6: Friends/relatives ever concerned with use**<br>Has a friend or relative or anyone else ever expressed concern about your use of tobacco products (cigarettes, chewing tobacco, cigars, e-cigarettes, etc.)?	Coded Fields:<br>1: No, Never<br>2: Yes, in the past 3 months<br>3: Yes, but not in the past 3 months	ATN 071 / Assist
**TOBACCO_7: Ever attempted to reduce/stop use**<br>Have you ever tried and failed to control, cut down or stop using tobacco products (cigarettes, chewing tobacco, cigars, e-cigarettes, etc.)?	Coded Fields:<br>1: No, Never<br>2: Yes, in the past 3 months<br>3: Yes, but not in the past 3 months	ATN 071 / Assist

Substance Use and Abuse		
**Characteristic**	**Format/Values**	**Source/ Comments**
**ALCOHOL_1: Lifetime use**<br>In your life, have you ever used alcoholic beverages (beer, wine, spirits, etc.)?<br><br>If **ALCOHOL_1=No** then skip to **CANNABIS_1**.	Coded Fields:<br>1: Yes<br>2: No	ATN 071 / Assist
**ALCOHOL_2: Use in past 3 months**<br>In the past 3 months, how often have you used alcoholic beverages (beer, wine, spirits, etc.)?<br><br>If **ALCOHOL_2 = Never** then skip **ALCOHOL_3-ALCOHOL_5**.	Coded Fields:<br>1: Never<br>2: Once or Twice<br>3. Monthly<br>4: Weekly<br>5: Daily or Almost Daily	ATN 071 / Assist
**ALCOHOL_2.1: Drinks in a typical day**<br>How many drinks containing alcohol do you have on a typical day?	Coded Fields:<br>1: 1 or 2<br>2: 3 or 4<br>3. 5 or 6<br>4: 7 to 9<br>5: 10 or more	AUDIT-C
**ALCOHOL_2.2: Binge Drinking**<br>How often do you have **[Insert Number]** or more drinks on one occasion?<br>**If participant sex assigned at birth is male, use 5 drinks. If female, use 4 drinks.**	Coded Fields:<br>1: Never<br>2: Less than monthly<br>3: Monthly<br>4: Weekly<br>5: Daily or almost daily	Customized version of AUDIT-C question. Reference: http://www.niaaa.nih.gov/alcohol-health/overview-alcohol-consumption/moderate-binge-drinking
**ALCOHOL_3: Desire to use in past 3 months**<br>During the past 3 months, how often have you had a desire to use alcoholic beverages (beer, wine, spirits, etc.)?	Coded Fields:<br>1: Never<br>2: Once or Twice<br>3. Monthly<br>4: Weekly<br>5: Daily or Almost Daily	ATN 071 / Assist
**ALCOHOL_4: Problems due to use in past 3 months**<br>During the past 3 months, how often has your use of alcoholic beverages (beer, wine, spirits, etc.) led to health, social, legal or financial problems?	Coded Fields:<br>1: Never<br>2: Once or Twice<br>3. Monthly<br>4: Weekly<br>5: Daily or Almost Daily	ATN 071 / Assist
**ALCOHOL_5: Failed to meet expectations in past 3 months**<br>During the past 3 months, how often have you failed to do what was normally expected of you because of your use of alcoholic beverages (beer, wine, spirits, etc.)?	Coded Fields:<br>1: Never<br>2: Once or Twice<br>3. Monthly<br>4: Weekly<br>5: Daily or Almost Daily	ATN 071 / Assist
**ALCOHOL_6: Friends/relatives ever concerned with use**<br>Has a friend or relative or anyone else ever expressed concern about your use of alcoholic beverages (beer, wine, spirits, etc.)?	Coded Fields:<br>1: No, Never<br>2: Yes, in the past 3 months<br>3: Yes, but not in the past 3 months	ATN 071 / Assist
**ALCOHOL_7: Ever attempted to reduce/stop use**<br>Have you ever tried and failed to control, cut down or stop using alcoholic beverages (beer, wine, spirits, etc.)?	Coded Fields:<br>1: No, Never<br>2: Yes, in the past 3 months<br>3: Yes, but not in the past 3 months	ATN 071 / Assist

Substance Use and Abuse		
**Characteristic**	**Format/Values**	**Source/ Comments**
**Cannabis Introductory Script:**<br>In this section, we will ask you about cannabis and synthetic cannabis use. Please report on all use even if you have a prescription or cannabis use is legal in your state or area.		
**CANNABIS_1: Lifetime use**<br>In your life, have you ever used cannabis (marijuana, pot, grass, hash, etc.) or synthetic cannabis?<br><br>If **CANNABIS_1=No** then skip to **COCAINE_1.**	Coded Fields:<br>1: Yes<br>2: No	ATN 071 / Assist
**CANNABIS_1.1**<br>Do you have or have you had a prescription to use cannabis (marijuana, pot, grass, hash, etc.) or synthetic cannabis?<br><br>If **CANNABIS_1.1 = No** then skip **CANNABIS_1.1.1.**	Coded Fields:<br>1: Yes<br>2: No	Added per SUA working group.
**CANNABIS_1.1.1**<br>Do you use or have you used cannabis (marijuana, pot, grass, hash, etc.) or synthetic cannabis for reasons other than prescription, or more frequently or at higher doses than prescribed?	Coded Fields:<br>1: Yes<br>2: No	
**CANNABIS_1.2**<br>At the time you last used cannabis and synthetic cannabis, was it legal to use in your state or area?	Coded Fields:<br>1: Yes<br>2: No<br>3. I don't know	Added per SUA working group.
**CANNABIS_2: Use in past 3 months**<br>In the past 3 months, how often have you used cannabis (marijuana, pot, grass, hash, etc.) or synthetic cannabis?<br><br>If **CANNABIS_2 = Never** then skip **CANNABIS_3-CANNABIS_5**.	Coded Fields:<br>1: Never<br>2: Once or Twice<br>3. Monthly<br>4: Weekly<br>5: Daily or Almost Daily	ATN 071 / Assist
**CANNABIS_3: Desire to use in past 3 months**<br>During the past 3 months, how often have you had a desire to use cannabis (marijuana, pot, grass, hash, etc.) or synthetic cannabis?	Coded Fields:<br>1: Never<br>2: Once or Twice<br>3. Monthly<br>4: Weekly<br>5: Daily or Almost Daily	ATN 071 / Assist
**CANNABIS_4: Problems due to use in past 3 months**<br>During the past 3 months, how often has your use of cannabis (marijuana, pot, grass, hash, etc.) or synthetic cannabis led to health, social, legal or financial problems?	Coded Fields:<br>1: Never<br>2: Once or Twice<br>3. Monthly<br>4: Weekly<br>5: Daily or Almost Daily	ATN 071 / Assist
**CANNABIS_5: Failed to meet expectations in past 3 months**<br>During the past 3 months, how often have you failed to do what was normally expected of you because of your use of cannabis (marijuana, pot, grass, hash, etc.) or synthetic cannabis?	Coded Fields:<br>1: Never<br>2: Once or Twice<br>3. Monthly<br>4: Weekly<br>5: Daily or Almost Daily	ATN 071 / Assist
**CANNABIS_6: Friends/relatives ever concerned with use**<br>Has a friend or relative or anyone else ever expressed concern about your use of cannabis (marijuana, pot, grass, hash, etc.) or synthetic cannabis?	Coded Fields:<br>1: No, Never<br>2: Yes, in the past 3 months<br>3: Yes, but not in the past 3 months	ATN 071 / Assist

Substance Use and Abuse		
**Characteristic**	**Format/Values**	**Source/ Comments**
**CANNABIS_7: Ever attempted to reduce/stop use**<br>Have you ever tried and failed to control, cut down or stop using cannabis (marijuana, pot, grass, hash, etc.) or synthetic cannabis?	Coded Fields:<br>1: No, Never<br>2: Yes, in the past 3 months<br>3: Yes, but not in the past 3 months	ATN 071 / Assist
**COCAINE_1: Lifetime use**<br>In your life, have you ever used cocaine (coke, crack, etc.)?<br><br>If **COCAINE_1=No** then skip to **AMPHETAMINE_1**.	Coded Fields:<br>1: Yes<br>2: No	ATN 071 / Assist
**COCAINE_2: Use in past 3 months**<br>In the past 3 months, how often have you used cocaine (coke, crack, etc.)?<br><br>If **COCAINE_2 = Never** then skip **COCAINE_3-COCAINE_5**.	Coded Fields:<br>1: Never<br>2: Once or Twice<br>3. Monthly<br>4: Weekly<br>5: Daily or Almost Daily	ATN 071 / Assist
**COCAINE_3: Desire to use in past 3 months**<br>During the past 3 months, how often have you had a desire to use cocaine (coke, crack, etc.)?	Coded Fields:<br>1: Never<br>2: Once or Twice<br>3. Monthly<br>4: Weekly<br>5: Daily or Almost Daily	ATN 071 / Assist
**COCAINE_4: Problems due to use in past 3 months**<br>During the past 3 months, how often has your use of cocaine (coke, crack, etc.) led to health, social, legal or financial problems?	Coded Fields:<br>1: Never<br>2: Once or Twice<br>3. Monthly<br>4: Weekly<br>5: Daily or Almost Daily	ATN 071 / Assist
**COCAINE_5: Failed to meet expectations in past 3 months**<br>During the past 3 months, how often have you failed to do what was normally expected of you because of your use of cocaine (coke, crack, etc.)?	Coded Fields:<br>1: Never<br>2: Once or Twice<br>3. Monthly<br>4: Weekly<br>5: Daily or Almost Daily	ATN 071 / Assist
**COCAINE_6: Friends/relatives ever concerned with use**<br>Has a friend or relative or anyone else ever expressed concern about your use of cocaine (coke, crack, etc.)?	Coded Fields:<br>1: No, Never<br>2: Yes, in the past 3 months<br>3: Yes, but not in the past 3 months	ATN 071 / Assist
**COCAINE_7: Ever attempted to reduce/stop use**<br>Have you ever tried and failed to control, cut down or stop using cocaine (coke, crack, etc.)?	Coded Fields:<br>1: No, Never<br>2: Yes, in the past 3 months<br>3: Yes, but not in the past 3 months	ATN 071 / Assist
**Amphetamine Introductory Script:**<br>In this section, we will ask you about amphetamine use. Please report on all non-prescription drug use. Please report on use of prescription amphetamines only if you have taken such medications for reasons other than prescription, or taken them more frequently or at higher doses than prescribed.		
**AMPHETAMINE_1: Lifetime use**<br>In your life, have you ever used amphetamine type stimulants (speed, meth, diet pills, ecstasy, Ritalin, Adderall, etc.)?<br><br>If **AMPHETAMINE_1=No** then skip to **INHALANTS_1**.	Coded Fields:<br>1: Yes<br>2: No	ATN 071 / Assist

Substance Use and Abuse		
**Characteristic**	**Format/Values**	**Source/ Comments**
**AMPHETAMINE_2: Use in past 3 months**<br>In the past 3 months, how often have you used amphetamine type stimulants (speed, meth, diet pills, ecstasy, Ritalin, Adderall, etc.)?<br><br>If **AMPHETAMINE_2 = Never** then skip **AMPHETAMINE_3-AMPHETAMINE_5**.	Coded Fields:<br>1: Never<br>2: Once or Twice<br>3. Monthly<br>4: Weekly<br>5: Daily or Almost Daily	ATN 071 / Assist
**AMPHETAMINE_3: Desire to use in past 3 months**<br>During the past 3 months, how often have you had a desire to use amphetamine type stimulants (speed, meth, diet pills, ecstasy, Ritalin, Adderall, etc.)?	Coded Fields:<br>1: Never<br>2: Once or Twice<br>3. Monthly<br>4: Weekly<br>5: Daily or Almost Daily	ATN 071 / Assist
**AMPHETAMINE_4: Problems due to use in past 3 months**<br>During the past 3 months, how often has your use of amphetamine type stimulants (speed, meth, diet pills, ecstasy, Ritalin, Adderall, etc.) led to health, social, legal or financial problems?	Coded Fields:<br>1: Never<br>2: Once or Twice<br>3. Monthly<br>4: Weekly<br>5: Daily or Almost Daily	ATN 071 / Assist
**AMPHETAMINE_5: Failed to meet expectations in past 3 months**<br>During the past 3 months, how often have you failed to do what was normally expected of you because of your use of amphetamine type stimulants (speed, meth, diet pills, ecstasy, Ritalin, Adderall, etc.)?	Coded Fields:<br>1: Never<br>2: Once or Twice<br>3. Monthly<br>4: Weekly<br>5: Daily or Almost Daily	ATN 071 / Assist
**AMPHETAMINE_6: Friends/relatives ever concerned with use**<br>Has a friend or relative or anyone else ever expressed concern about your use of amphetamine type stimulants (speed, meth, diet pills, ecstasy, Ritalin, Adderall, etc.)?	Coded Fields:<br>1: No, Never<br>2: Yes, in the past 3 months<br>3: Yes, but not in the past 3 months	ATN 071 / Assist
**AMPHETAMINE_7: Ever attempted to reduce/stop use**<br>Have you ever tried and failed to control, cut down or stop using amphetamine type stimulants (speed, meth, diet pills, ecstasy, Ritalin, Adderall, etc.)?	Coded Fields:<br>1: No, Never<br>2: Yes, in the past 3 months<br>3: Yes, but not in the past 3 months	ATN 071 / Assist
**INHALANTS_1: Lifetime use**<br>In your life, have you ever used inhalants (nitrous, glue, petrol, paint thinner, etc.)?<br><br>If **INHALANTS_1=No** then skip to **SEDATIVES_1**.	Coded Fields:<br>1: Yes<br>2: No	ATN 071 / Assist
**INHALANTS_2: Use in past 3 months**<br>In the past 3 months, how often have you used inhalants (nitrous, glue, petrol, paint thinner, etc.)?<br><br>If **INHALANTS_2 = Never** then skip **INHALANTS_3-INHALANTS_5**.	Coded Fields:<br>1: Never<br>2: Once or Twice<br>3. Monthly<br>4: Weekly<br>5: Daily or Almost Daily	ATN 071 / Assist
**INHALANTS_3: Desire to use in past 3 months**<br>During the past 3 months, how often have you had a desire to use inhalants (nitrous, glue, petrol, paint thinner, etc.)?	Coded Fields:<br>1: Never<br>2: Once or Twice<br>3. Monthly<br>4: Weekly<br>5: Daily or Almost Daily	ATN 071 / Assist

Substance Use and Abuse		
**Characteristic**	**Format/Values**	**Source/ Comments**
**INHALANTS_4: Problems due to use in past 3 months**<br>During the past 3 months, how often has your use of inhalants (nitrous, glue, petrol, paint thinner, etc.) led to health, social, legal or financial problems?	Coded Fields:<br>1: Never<br>2: Once or Twice<br>3. Monthly<br>4: Weekly<br>5: Daily or Almost Daily	ATN 071 / Assist
**INHALANTS_5: Failed to meet expectations in past 3 months**<br>During the past 3 months, how often have you failed to do what was normally expected of you because of your use of inhalants (nitrous, glue, petrol, paint thinner, etc.)?	Coded Fields:<br>1: Never<br>2: Once or Twice<br>3. Monthly<br>4: Weekly<br>5: Daily or Almost Daily	ATN 071 / Assist
**INHALANTS_6: Friends/relatives ever concerned with use**<br>Has a friend or relative or anyone else ever expressed concern about your use of inhalants (nitrous, glue, petrol, paint thinner, etc.)?	Coded Fields:<br>1: No, Never<br>2: Yes, in the past 3 months<br>3: Yes, but not in the past 3 months	ATN 071 / Assist
**INHALANTS_7: Ever attempted to reduce/stop use**<br>Have you ever tried and failed to control, cut down or stop using inhalants (nitrous, glue, petrol, paint thinner, etc.)?	Coded Fields:<br>1: No, Never<br>2: Yes, in the past 3 months<br>3: Yes, but not in the past 3 months	ATN 071 / Assist
**Sedative Introductory Script:**<br>In this section, we will ask you about sedative, tranquilizer, and sleeping pills use. Please report on all non-prescription drug use. Please report on use of prescription drugs only if you have taken such medications for reasons other than prescription, or taken them more frequently or at higher doses than prescribed.		
**SEDATIVES_1: Lifetime use**<br>In your life, have you ever used sedatives, tranquilizers, or sleeping pills (Valium, Serepax, Rohypnol, Xanax, Ambien, GHB, etc.)?<br><br>If **SEDATIVES_1=No** then skip to **HALLUCINOGENS_1**.	Coded Fields:<br>1: Yes<br>2: No	ATN 071 / Assist
**SEDATIVES_2: Use in past 3 months**<br>In the past 3 months, how often have you used sedatives, tranquilizers, or sleeping pills (Valium, Serepax, Rohypnol, Xanax, Ambien, GHB, etc.)?<br><br>If **SEDATIVES_2 = Never** then skip **SEDATIVES_3-SEDATIVES_5**.	Coded Fields:<br>1: Never<br>2: Once or Twice<br>3. Monthly<br>4: Weekly<br>5: Daily or Almost Daily	ATN 071 / Assist
**SEDATIVES_3: Desire to use in past 3 months**<br>During the past 3 months, how often have you had a desire to use sedatives, tranquilizers, or sleeping pills (Valium, Serepax, Rohypnol, Xanax, Ambien, GHB, etc.)?	Coded Fields:<br>1: Never<br>2: Once or Twice<br>3. Monthly<br>4: Weekly<br>5: Daily or Almost Daily	ATN 071 / Assist
**SEDATIVES_4: Problems due to use in past 3 months**<br>During the past 3 months, how often has your use of sedatives, tranquilizers, or sleeping pills (Valium, Serepax, Rohypnol, Xanax, Ambien, GHB, etc.) led to health, social, legal or financial problems?	Coded Fields:<br>1: Never<br>2: Once or Twice<br>3. Monthly<br>4: Weekly<br>5: Daily or Almost Daily	ATN 071 / Assist
**SEDATIVES_5: Failed to meet expectations in past 3 months**<br>During the past 3 months, how often have you failed to do what was normally expected of you because of your use of sedatives, tranquilizers, or sleeping pills (Valium, Serepax, Rohypnol, Xanax, Ambien, GHB, etc.)?	Coded Fields:<br>1: Never<br>2: Once or Twice<br>3. Monthly<br>4: Weekly<br>5: Daily or Almost Daily	ATN 071 / Assist

Substance Use and Abuse		
**Characteristic**	**Format/Values**	**Source/ Comments**
**SEDATIVES_6: Friends/relatives ever concerned with use**<br>Has a friend or relative or anyone else ever expressed concern about your use of sedatives, tranquilizers, or sleeping pills (Valium, Serepax, Rohypnol, Xanax, Ambien, GHB, etc.)?	Coded Fields:<br>1: No, Never<br>2: Yes, in the past 3 months<br>3: Yes, but not in the past 3 months	ATN 071 / Assist
**SEDATIVES_7: Ever attempted to reduce/stop use**<br>Have you ever tried and failed to control, cut down or stop using sedatives, tranquilizers, or sleeping pills (Valium, Serepax, Rohypnol, Xanax, Ambien, GHB, etc.)?	Coded Fields:<br>1: No, Never<br>2: Yes, in the past 3 months<br>3: Yes, but not in the past 3 months	ATN 071 / Assist
**HALLUCINOGENS_1: Lifetime use**<br>In your life, have you ever used hallucinogens (LSD, acid, mushrooms, PCP, Ketamine, etc.)?<br><br>If **HALLUCINOGENS_1=No** then skip to **OPIOIDS_1.**	Coded Fields:<br>1: Yes<br>2: No	ATN 071 / Assist
**HALLUCINOGENS_2: Use in past 3 months**<br>In the past 3 months, how often have you used hallucinogens (LSD, acid, mushrooms, PCP, Ketamine, etc.)?<br><br>If **HALLUCINOGENS_2 = Never**<br>then skip **HALLUCINOGENS_3-HALLUCINOGENS_5.**	Coded Fields:<br>1: Never<br>2: Once or Twice<br>3. Monthly<br>4: Weekly<br>5: Daily or Almost Daily	ATN 071 / Assist
**HALLUCINOGENS_3: Desire to use in past 3 months**<br>During the past 3 months, how often have you had a desire to use hallucinogens (LSD, acid, mushrooms, PCP, Ketamine, etc.)?	Coded Fields:<br>1: Never<br>2: Once or Twice<br>3. Monthly<br>4: Weekly<br>5: Daily or Almost Daily	ATN 071 / Assist
**HALLUCINOGENS_4: Problems due to use in past 3 months**<br>During the past 3 months, how often has your use of hallucinogens (LSD, acid, mushrooms, PCP, trips, Ketamine, etc.) led to health, social, legal or financial problems?	Coded Fields:<br>1: Never<br>2: Once or Twice<br>3. Monthly<br>4: Weekly<br>5: Daily or Almost Daily	ATN 071 / Assist
**HALLUCINOGENS_5: Failed to meet expectations in past 3 months**<br>During the past 3 months, how often have you failed to do what was normally expected of you because of your use of hallucinogens (LSD, acid, mushrooms, PCP, Ketamine, etc.)?	Coded Fields:<br>1: Never<br>2: Once or Twice<br>3. Monthly<br>4: Weekly<br>5: Daily or Almost Daily	ATN 071 / Assist
**HALLUCINOGENS_6: Friends/relatives ever concerned with use**<br>Has a friend or relative or anyone else ever expressed concern about your use of hallucinogens (LSD, acid, mushrooms, PCP, Ketamine, etc.)?	Coded Fields:<br>1: No, Never<br>2: Yes, in the past 3 months<br>3: Yes, but not in the past 3 months	ATN 071 / Assist
**HALLUCINOGENS_7: Ever attempted to reduce/stop use**<br>Have you ever tried and failed to control, cut down or stop using hallucinogens (LSD, acid, mushrooms, PCP, Ketamine, etc.)?	Coded Fields:<br>1: No, Never<br>2: Yes, in the past 3 months<br>3: Yes, but not in the past 3 months	ATN 071 / Assist
**Opioid Introductory Script:**<br>In this section, we will ask you about opioid use. Please report on use of all non-prescription drugs. Please report on use of prescription drugs only if you have taken such medications for reasons other than prescription, or taken them more frequently or at higher doses than prescribed.		
**OPIOIDS_1: Lifetime use**<br>In your life, have you ever used opioids (heroin, morphine, methadone, codeine, Oxycontin, Percocet, Vicodin, etc.)?<br><br>If **OPIOIDS_1=No** then skip to **END.**	Coded Fields:<br>1: Yes<br>2: No	ATN 071 / Assist

Substance Use and Abuse		
**Characteristic**	**Format/Values**	**Source/ Comments**
**OPIOIDS_2: Use in past 3 months**<br>In the past 3 months, how often have you used opioids (heroin, morphine, methadone, codeine, Oxycontin, Percocet, Vicodin, etc.)?<br><br>If **OPIOIDS_2 = Never** then skip **OPIOIDS_2.1-OPIOIDS_5**.	Coded Fields:<br>1: Never<br>2: Once or Twice<br>3. Monthly<br>4: Weekly<br>5: Daily or Almost Daily	ATN 071 / Assist
**OPIOIDS_2.1: Heroin use in the past 3 months**<br>In the past 3 months, has your opioid use included heroin?<br><br>If **OPIOIDS_2.1 = No** then skip **OPIOIDS_2.2**.	Coded Fields:<br>1: Yes<br>2: No	
**OPIOIDS_2.2: Heroin as primary opioid**<br>In the past 3 months, was heroin the most common opioid that you used?	Coded Fields:<br>1: Yes<br>2: No	
**OPIOIDS_3: Desire to use in past 3 months**<br>During the past 3 months, how often have you had a desire to use opioids (heroin, morphine, methadone, codeine, Oxycontin, Percocet, Vicodin, etc.)?	Coded Fields:<br>1: Never<br>2: Once or Twice<br>3. Monthly<br>4: Weekly<br>5: Daily or Almost Daily	ATN 071 / Assist
**OPIOIDS_4: Problems due to use in past 3 months**<br>During the past 3 months, how often has your use of opioids (heroin, morphine, methadone, codeine, Oxycontin, Percocet, Vicodin, etc.) led to health, social, legal or financial problems?	Coded Fields:<br>1: Never<br>2: Once or Twice<br>3. Monthly<br>4: Weekly<br>5: Daily or Almost Daily	ATN 071 / Assist
**OPIOIDS_5: Failed to meet expectations in past 3 months**<br>During the past 3 months, how often have you failed to do what was normally expected of you because of your use of opioids (heroin, morphine, methadone, codeine, Oxycontin, Percocet, Vicodin, etc.)?	Coded Fields:<br>1: Never<br>2: Once or Twice<br>3. Monthly<br>4: Weekly<br>5: Daily or Almost Daily	ATN 071 / Assist
**OPIOIDS_6: Friends/relatives ever concerned with use**<br>Has a friend or relative or anyone else ever expressed concern about your use of opioids (heroin, morphine, methadone, codeine, Oxycontin, Percocet, Vicodin, etc.)?	Coded Fields:<br>1: No, Never<br>2: Yes, in the past 3 months<br>3: Yes, but not in the past 3 months	ATN 071 / Assist
**OPIOIDS_7: Ever attempted to reduce/stop use**<br>Have you ever tried and failed to control, cut down or stop using opioids (heroin, morphine, methadone, codeine, Oxycontin, Percocet, Vicodin, etc.)?	Coded Fields:<br>1: No, Never<br>2: Yes, in the past 3 months<br>3: Yes, but not in the past 3 months	ATN 071 / Assist

Table 4: ATN Standard Domain: HIV Negative Cascade

HIV Negative Cascade		
**Characteristic**	**Format/Values**	**Comments**
**Lifetime HIV Testing**<br>Have you ever had an HIV test?	Coded Fields:<br>1. Yes<br>2. No	**Source:** NHBS
*Ask if **Lifetime HIV Testing** is **Yes***<br><br>**First HIV Test Date**<br>When did you have your first HIV test?<br>*Please enter the month and year.*	MMYYYY	**Source:** NHBS
*Ask if **Lifetime HIV Testing** is **Yes***<br><br>**Ever Tested HIV Positive**<br>Have you ever tested positive for HIV? (Do you have HIV?)<br><br>*This includes having gotten the virus earlier, but are now suppressed (undetectable).*	Coded Fields:<br>1. Yes<br>2. No	**Source:** NHBS
*Ask if **Lifetime HIV Testing** is **Yes***<br><br>**Most Recent HIV Test Date**<br>When did you have your most recent HIV test?<br>*Please enter the month and year.*	MMYYYY	**Source:** NHBS<br>Include logic check: Date should not be earlier than First HIV Test Date.
*Ask if **Lifetime HIV Testing** is **Yes***<br>**Most Recent Test Result**<br>What was the result of your most recent HIV test?	Coded Fields:<br>1. Negative, you do NOT have HIV<br>2. Positive, you DO have HIV<br>3. Indeterminate<br>4. Never obtained results<br><br>5. Have not yet been provided results	**Source:** NHBS
Ask if **Most Recent Test Result** *is **Positive***<br><br>**Most Recent First Positive**<br>Was your most recent test your first positive test?	Coded Fields:<br>1. Yes<br>2. No	**Source:** NHBS
**Lifetime STI Testing**<br>A sexually transmitted infection (STI) is an infection transmitted through sexual activity such as syphilis, gonorrhea, chlamydia, herpes, or genital warts.<br><br>Have you ever been tested for an STI that was not HIV?	Coded Fields:<br>1. Yes<br>2. No	**Source:** Element
*Ask if **Lifetime STI Testing** is **Yes***<br><br>**STI Testing 12 months**<br>In the past 12 months, have you been tested for an STI that was not HIV?	Coded Fields:<br>1. Yes<br>2. No	**Source:** Element

HIV Negative Cascade		
**Characteristic**	**Format/Values**	**Comments**
**STI Diagnosis 12 months**<br>Please indicate whether you have been diagnosed with any of the following sexually transmitted infections in the past 12 months<br>*Please select all that apply.*	Coded Fields:<br>1. Chlamydia<br>2. Genital warts, anal warts, HPV<br>3. Gonorrhea<br>4. Hepatitis B<br>5. Hepatitis C<br>6. Herpes, HSV1/HSV2<br>7. Syphilis<br>8. Urethritis	**Source:** Scale It Up
**Post-Exposure Prophylaxis (PEP) Awareness**<br>Before today, have you ever heard of people taking anti-HIV medicines AFTER a sexual or drug use exposure, to reduce the risk of getting HIV? This is called post-exposure prophylaxis, or PEP.	Coded Field:<br>1. Yes<br>2. No	**Source:** UCSF Risk Assessment
**Lifetime PEP Use**<br>Have you ever taken post-exposure prophylaxis (PEP) AFTER a sexual or drug use exposure, to reduce the risk of getting HIV?	Coded Field:<br>1. Yes<br>2. No	**Source:** UCSF Risk Assessment with wording changed to match NHBS PrEP question
*Ask if **Lifetime PEP Use** is **Yes***<br>**PEP Use 12 Months**<br>In the past 12 months, have you taken PEP (post-exposure prophylaxis) AFTER a sexual or drug use exposure, to reduce the risk of getting HIV?	Coded Field:<br>1. Yes<br>2. No	**Source:** NHBS
**Pre-Exposure Prophylaxis (PrEP) Awareness**<br>Before today, have you ever heard of people regularly taking anti-HIV medicines BEFORE a sexual or drug use exposure, to reduce the risk of getting HIV? This is called pre-exposure prophylaxis, or PrEP.	Coded Field:<br>1. Yes<br>2. No	**Source:** NHBS
**Lifetime PrEP Use**<br>Have you ever taken PrEP (pre-exposure prophylaxis) BEFORE a sexual or drug use exposure, to reduce the risk of getting HIV?	Coded Field:<br>1. Yes, I am on PrEP right now<br>2. Yes, I was in the past, but I'm not on PrEP anymore<br>3. No, I've never taken PrEP	**Source:** Scale It Up - *Stage 4b: PrEP Action*
*Ask if **Lifetime PrEP Use** is **Yes***<br>**How Obtained PrEP**<br>Did you get your PrEP from the following people or places?<br>*Please select all that apply.*	Coded Field:<br>**1.** Doctor or other health care provider<br>**2.** Sex partner, friend, relative, or acquaintance<br>**3.** Internet<br>**4.** Other, please specify: ____	**Source:** NHBS

HIV Negative Cascade		
**Characteristic**	**Format/Values**	**Comments**
Ask if ***Lifetime PrEP Use*** *is* ***Yes***<br><br>**First PrEP Use Date**<br><br>When did you first begin taking PrEP?	MMYYYY	**Source:** Scale It Up
Ask if ***Lifetime PrEP Use*** *is* ***Yes, I am <u>on PrEP right now</u>***<br><br>**PrEP Adherence**<br>While looking at the scale below, what percent of time did you take your PrEP medication as prescribed in the past 7 days?<br><br>*0% would mean 'NONE' of the time and 100% would mean 'ALL' of the time. If you are unsure, make a best guess.*<br><br>Visual Analog Scale:<br>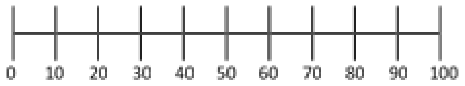	Coded Field:<br>1. 0%<br>2. 10%<br>3. 20%<br>4. 30%<br>5. 40%<br>6. 50%<br>7. 60%<br>8. 70%<br>9. 80%<br>10. 90%<br>11. 100%	**Source:** CARES
Ask if ***Lifetime PrEP Use*** *is* ***Yes***<br><br>**Barriers to Regular PrEP Use**<br>What has gotten in the way of you taking your regular PrEP doses?<br><br>*Please select all that apply.*	Coded Field:<br>1. I have not had any trouble taking my regular PrEP doses<br>2. My work/school schedule got in the way<br>3. I forgot<br>4. I did not think I needed it anymore<br>5. I had difficulty getting the medication<br>6. I had side effects that made me sick<br>7. My friends or family did not support me taking PrEP<br>8. Other, specify ________	**Source:** CARES, Scale It Up

HIV Negative Cascade		
**Characteristic**	**Format/Values**	**Comments**
Ask if ***Lifetime PrEP Use*** *is* ***No, I have never take PrEP***<br><br>**Barriers to PrEP Uptake**<br>What are your reasons for not starting PrEP?<br><br>*Please select all that apply.*	Coded Field:<br>1. I had not heard about PrEP until today<br>2. My work/school schedule could get in the way<br>3. I would forget<br>4. I don't think I need it<br>5. I would have difficulty getting the medication<br>6. I am nervous about side effects that might make me sick<br>7. My friends or family would not support me taking PrEP<br>8. Other, specify _________	**Source:** Adapted from previous question for those who never used PrEP.

Table 5: ATN Standard Domain: HIV Positive Cascade Participant Characteristics

**HIV Positive Cascade Participant Characteristics**		
**Characteristic**	**Format/Values**	**Comments**
**Date of HIV Diagnosis**<br>When did you first test positive for HIV?<br>*Please provide the month and year.*	MMYYYY	**Source:** NHBS<br>**Cascade:** Diagnosis
**Ever Seen Provider for HIV**<br>Have you ever been seen by a doctor, nurse, or other health care provider for a medical evaluation or care related to your HIV infection?	Coded Field:<br>1. Yes<br>2. No	**Source:** NHBS<br>**Cascade:** Receipt of Care
*Ask if **Ever Seen Provider for HIV** is **Yes***<br><br>**Date First Saw Provider After HIV+**<br>When did you first go to a health care provider after learning you had HIV?<br>*Please provide the month and year.*	MMYYYY	**Source:** NHBS<br>**Cascade:** Linked to Care<br><br>Include logic check: Date should not be earlier than the Date of HIV Diagnosis.
*Ask if **Ever Seen Provider for HIV** is **Yes***<br><br>**Most Recent HIV Care**<br>When did you last go to a health care provider for HIV care?<br>*Please provide the month and year.*	MMYYYY	**Source:** NHBS<br>**Cascade:** Retained in Care<br>Include logic check: Date should not be earlier than the Date First Saw Provider After HIV+.
*Ask if **Ever Seen Provider for HIV** is **Yes***<br><br>**History of Not Seeing Provider**<br>Was there a time in the past after you first engaged in HIV care that you went 6 months or longer without a medical visit with your HIV provider?	Coded Field:<br>1. Yes<br>2. No	**Source:** Developed by HPC-WG<br>**Cascade:** Retained in Care
**Schedule Appointments Past 12 Months**<br>In the last 12 months, how many scheduled appointments did you have?<br><br>*By scheduled appointments, we mean routine appointments (not walk-in or urgent care appointments for acute/urgent issue) that were planned and booked, whether you attended them or not.*	Integer	**Source:** ATN 132 Epic Allies<br>**Cascade:** Retained in Care
*Ask if **Schedule Appointments Past 12 Months** is **1 or more***<br><br>**Missed Appointments Past 12 Months**<br>In last 12 months, how many of your scheduled appointments did you miss because you didn't show or forgot?<br><br>*By scheduled appointments, we mean routine appointments, not walk-in or urgent care appointments for acute/urgent issue.*	Integer	**Source:** ATN 132 Epic Allies<br>**Cascade:** Retained in Care<br>Include logic check: Response should not be greater than the reported number of Scheduled Appointments in Past 12 Months.

HIV Positive Cascade Participant Characteristics		
**Characteristic**	**Format/Values**	**Comments**
*Ask if **Missed Appointments Past 12 Months** is **1 or more***<br><br>**Last Time Appointment Missed**<br>When was the last time you missed a scheduled appointment for HIV medical care? Would you say:<br><br>*By scheduled appointments, we mean routine appointments, not walk-in or urgent care appointments for acute/urgent issue.*	Coded Field:<br>1. Within the last month<br>2. 2 months ago<br>3. 3 months ago<br>4. 4 months ago<br>5. 5 months ago<br>6. 6 months ago<br>7. 6 or more months ago	**Source:** ATN 132 Epic Allies<br>**Cascade:** Retained in Care
**Future HIV Appointment**<br>Do you have a scheduled visit with your HIV provider in the next 6 months?<br><br>*By scheduled appointments, we mean routine appointments, not walk-in or urgent care appointments for acute/urgent issue.*	Coded Field:<br>1. Yes<br>2. No	**Source:** Developed by HPC-WG<br>**Cascade:** Retained in Care
*Ask if **Future HIV Appointment** is **Yes***<br><br>**Date of Future HIV Appointment**<br>What month and year is the visit with your HIV provider scheduled?	MMYYYY	**Source:** Developed by HPC-WG<br>**Cascade:** Retained in Care<br>Include logic check: Date should be after the current date.
**Ever HIV Viral Load Test**<br>Have you ever had an HIV viral load (VL) test?	Coded Field:<br>1. Yes<br>2. No	**Source:** NHBS<br>**Cascade:** Viral Suppression
*Ask if **Ever HIV Viral Load Test** is **Yes***<br><br>**Recent HIV VL Test Date**<br>What month and year was your most recent HIV viral load test?<br>*Please provide the month and year.*	MMYYYY	**Source:** NHBS<br>**Cascade:** Viral Suppression<br>Include logic check: Date should not be after current date.
*Ask if **Ever HIV Viral Load Test** is **Yes***<br><br>**HIV Viral Load Test Results**<br>What was the result of your most recent viral load test?<br><br>*Please select one of the answers that most closely matches your last viral load test results in viral copies/ml.*	Coded Field:<br>1. Below the level of detection, undetectable<br>2. Detectable, but less than 5,000<br>3. 5,000 – 10,000<br>4. Greater than 10,000<br>5. Don't know	**Source:** NHBS<br>**Cascade:** Viral Suppression
**Ever CD4 Count**<br>Have you ever had a CD4 count test?	Coded Field:<br>1. Yes<br>2. No	**Source:** ATN 132 Epic Allies<br>**Cascade:** Receipt of Care

HIV Positive Cascade Participant Characteristics		
**Characteristic**	**Format/Values**	**Comments**
*Ask if **Ever CD4 Count** is **Yes***<br><br>**Recent CD4 Count Test Date**<br>What month and year was your most recent CD4 count test?<br>*Please provide the month and year.*	MMYYYY	**Source:** Developed by HPC-WG to follow flow of viral load measures<br>**Cascade:** Receipt of Care<br>Include logic check: Date should not be after current date.
*Ask if **Ever CD4 Count** is **Yes***<br><br>**Recent CD4 Count Results**<br>What was the result of your most recent CD4 count test?<br><br>*Please select one of the answers that most closely matches your most recent CD4 count results in cells/mm^3.*	Coded Field:<br>1. Less than 50<br>2. 51 – 200<br>3. 201 – 350<br>4. 351 – 500<br>5. Greater than 500<br>6. Don't know	**Source:** ATN 132 Epic Allies<br>**Cascade:** Receipt of Care
**Current ARV Treatment**<br>Are you currently taking pills or other medicines to treat your HIV (antiretroviral (ARV) medications, not medications you are taking to prevent other complications of HIV)?	Coded Field:<br>**1.** Yes<br>**2.** No	**Source:** ATN 132 Epic Allies<br>**Cascade:** ART uptake
*Ask if **Current ARV Treatment** is **Yes***<br><br>**Date Started ARV Treatment**<br>What was the month and year you first started taking HIV medications?	MMYYYY	**Source:** ATN138 Y-TWM<br>**Cascade:** ART uptake<br>Include logic check: Date should not be earlier than the Date of HIV Diagnosis.
*Ask if **Current ARV Treatment** is **Yes***<br><br>**Number of ARV Doses Prescribed**<br>How many times during the day has your HIV provider told you to take a dose of medicine (pills or other medicines) to treat your HIV?	Coded Field:<br>**1.** 1 time a day<br>**2.** 2 times a day<br>**3.** 3 or more times a day	**Source:** ATN 132 Epic Allies<br>**Cascade:** ART uptake
*Ask if **Current ARV Treatment** is **Yes***<br><br>**Percent of HIV Medication Taken**<br>In the last 7 days, what percent of your prescribed HIV medication have you taken?<br><br>*0% means you have taken no medication, 50% means you have taken half your medication, 100% means you have taken every single dose of your medication. If you are unsure, make a best guess.*<br><br>Visual Analog Scale:<br>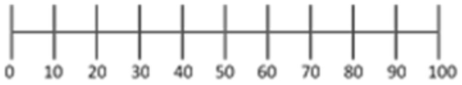	Coded Field:<br>**1.** 0%<br>**2.** 10%<br>**3.** 20%<br>**4.** 30%<br>**5.** 40%<br>**6.** 50%<br>**7.** 60%<br>**8.** 70%<br>**9.** 80%<br>**10.** 90%<br>**11.** 100%	**Source:** ATN138 Y-TWM (adjusted time period to fit validated literature)<br>**Cascade:** ART Adherence<br><br>Visual Analog scale (VAS) – Validated self-report measure of medication adherence.

HIV Positive Cascade Participant Characteristics		
**Characteristic**	**Format/Values**	**Comments**
*Ask if **Current ARV Treatment** is **Yes***<br><br>**Missed All HIV Medication Doses**<br>During the past 4 days, on how many days have you missed taking all your HIV medication doses?	Coded Fields:<br>**1.** None<br>**2.** 1 day<br>**3.** 2 days<br>**4.** 3 days<br>**5.** 4 days	**Source:** ATN138 Y-TWM<br>**Cascade:** ART Adherence
*Ask if **Current ARV Treatment** is **Yes***<br><br>**Missed at Least 1 HIV Medication Dose**<br>In the last 30 days, on how many days did you miss at least one dose of any of your HIV medicines?	Integer (0-30)	**Source:** ATN138 Y-TWM<br>**Cascade:** ART Adherence<br>Include logic check: Response must exceed the response to number of days Missed All HIV Medication Doses.

Table 6: ATN Standard Domain: HIV Positive Cascade Biomedical Data

**HIV Positive Cascade Biomedical Data**		
**Characteristic**	**Format/Values**	**Comments**
Instructions:<br>Source documentation by laboratory report should be in the medical records, but if not present, support for a viral load (VL) result via a clinician's note or verbal report by a clinician or subject will be accepted as long as the source is indicated on this form. As part of best clinical practice, sites should try to obtain the most definitive source verification and should use that as the source for documentation for this study.<br><br>If multiple source documents are available to substantiate the VL results, record the most definitive data source using the following hierarchical order: (1) laboratory report (2) clinician's note (3) verbal report by a clinician (4) verbal report by the subject.		
**Biomed HIV Viral Load (VL)**<br>Does the subject have at least one viral load (VL) result?	Coded Field:<br>1. Yes<br>2. No	**Source:** ATN125<br>**Cascade:** Viral Suppression
*Ask if **Biomed HIV Viral Load (VL)** is **Yes***<br><br>**HIV VL Collection Date**<br>What date was the blood collected for viral load testing?	MMDDYYYY	**Source:** ATN125<br>**Cascade:** Viral Suppression
*Ask if **Biomed HIV Viral Load (VL)** is **Yes***<br><br>**Viral Load Result**<br>What was the viral load result in copies/ml?<br><br>*Record the numerical value of the result in the spaces provided and check the symbol that describes what the numerical value represents, whether equal to, less than or greater than the recorded value. For a result that is reported as "below the lower limit of detection," record the assay's lower limit of detection as the numerical value and check the "less than (<)" symbol. For a result reported as "above the upper limit of quantitation," record the assay's upper limit of quantitation as the numerical value and check the "greater than (>)" symbol.*	Integer (length=10)<br><br>Coded Field:<br>1. Equal to (=)<br>2. Less than (<)<br>3. Greater than (>)	**Source:** ATN125<br>**Cascade:** Viral Suppression<br><br>Note: This measure requires two response fields.

HIV Positive Cascade Biomedical Data		
**Characteristic**	**Format/Values**	**Comments**
*Ask if **Biomed HIV Viral Load (VL)** is **Yes***<br><br>**Viral Load Assay Type**<br>What type of assay was used?	Coded Field:<br>1. Versant HIV-1 RNA 3.0 (bDNA) (Bayer/Siemans)<br>2. NucliSENS® HIV-1 QT (NASBA) (OrganonTeknika/bioMerieux)<br>3. Roche Amplicor® HIV-1 Monitor – Standard<br>4. Roche Amplicor® ™ HIV-1 Monitor – Ultrasensitive<br>5. Roche COBAS AmpliPrep/COBAS® TaqMan® HIV-1 Test, v1.0<br>6. Abbott RealTime™ HIV-1 Assay<br>7. NucliSENS® EasyQ HIV-1 Assay v1.0 or v2.0 (OrganonTeknika/bioMerieux)<br>8. Roche COBAS AmpliPrep/COBAS® TaqMan® HIV-1 Test, v2.0<br>9. Quantiplex HIV-1 RNA 3.0 (bDNA) (Chiron)<br>10. Unknown<br>11. Other, specify	**Source:** ATN125<br>**Cascade:** Viral Suppression
*Ask if **Biomed HIV Viral Load (VL)** is **Yes***<br><br>**Viral Load Assay Lower Limit**<br>What is the assay's lower limit of detection (in copies/ml)?	Integer (length=5)	**Source:** ATN125<br>**Cascade:** Viral Suppression
*Ask if **Biomed HIV Viral Load (VL)** is **Yes***<br><br>**Viral Load Assay Upper Limit**<br>What is the assay's upper limit of detection?	Integer (length=5)	**Source:** ATN125<br>**Cascade:** Viral Suppression
*Ask if **Biomed HIV Viral Load (VL)** is **Yes***<br><br>**Viral Load Data Source**<br>What is the data source of the viral load results?	Coded Field:<br>1. Laboratory report<br>2. Clinician's notes<br>3. Verbal report by clinician<br>4. Verbal report by subject<br>5. Other	**Source:** ATN125<br>**Cascade:** Viral Suppression

For studies collecting CD4 count from biomedical data, the following is recommended to be collected:

- CD4 Collection Date,
- CD4+ T-Cell Absolute COUNT (cells/mm^3),
- CD4+ T-Cell Percent (%), and
- Data Source (similar to Viral Load Data Source above)

Table 7: ATN Additional Measures: Mental Health

Mental Health		
**Characteristic**	**Format/Values**	**Comments**
**Introduction to PHQ-8:**<br>Over the past 2 weeks, how often have you been bothered by any of the following problems?		
**PHQ8_Q1**<br>Little interest or pleasure in doing things?	Coded Fields:<br>1. Not at all<br>2. Several days<br>3. More than half the days<br>4. Nearly every day	**Source:** iTech specific (PHQ-2 Screeener Q1/ PHQ-8)
**PHQ8_Q2**<br>Feeling down, depressed, or hopeless?	Coded Fields:<br>1. Not at all<br>2. Several days<br>3. More than half the days<br>4. Nearly every day	**Source:** iTech specific (PHQ-2 Screeener Q2/ PHQ-8)
*Ask if **PHQ8_Q1 + PHQ8_Q2** ≥ 3*<br>**PHQ8_Q3**<br>Trouble falling or staying asleep, or sleeping too much?	Coded Fields:<br>1. Not at all<br>2. Several days<br>3. More than half the days<br>4. Nearly every day	**Source:** iTech specific (PHQ-8)
*Ask if **PHQ8_Q1 + PHQ8_Q2** ≥ 3*<br>**PHQ8_Q4**<br>Feeling tired or having little energy?	Coded Fields:<br>1. Not at all<br>2. Several days<br>3. More than half the days<br>4. Nearly every day	**Source:** iTech specific (PHQ-8)
*Ask if **PHQ8_Q1 + PHQ8_Q2** ≥ 3*<br>**PHQ8_Q5**<br>Poor appetite or overeating?	Coded Fields:<br>1. Not at all<br>2. Several days<br>3. More than half the days<br>4. Nearly every day	**Source:** iTech specific (PHQ-8)
*Ask if **PHQ8_Q1 + PHQ8_Q2** ≥ 3*<br>**PHQ8_Q6**<br>Feeling bad about yourself - or that you are a failure or have let yourself or your family down?	Coded Fields:<br>1. Not at all<br>2. Several days<br>3. More than half the days<br>4. Nearly every day	**Source:** iTech specific (PHQ-8)
*Ask if **PHQ8_Q1 + PHQ8_Q2** ≥ 3*<br>**PHQ8_Q7**<br>Trouble concentrating on things, such as reading the newspaper or watching television?	Coded Fields:<br>1. Not at all<br>2. Several days<br>3. More than half the days<br>4. Nearly every day	**Source:** iTech specific (PHQ-8)

Mental Health		
**Characteristic**	**Format/Values**	**Comments**
*Ask if **PHQ8_Q1 + PHQ8_Q2 ≥ 3***<br><br>**PHQ8_Q8**<br>Moving or speaking so slowly that other people could have noticed, or the opposite - being so fidgety or restless that you have been moving around a lot more than usual?	Coded Fields:<br>1. Not at all<br>2. Several days<br>3. More than half the days<br>4. Nearly every day	**Source:** iTech specific (PHQ-8)
**Introduction to GAD-7:**<br>Over the past 2 weeks, how often have you been bothered by any of the following problems?		
**GAD7_Q1**<br>Feeling nervous, anxious or on edge?	Coded Fields:<br>1. Not at all<br>2. Several days<br>3. More than half the days<br>4. Nearly every day	**Source:** iTech specific (GAD-2 Screener Q1/ GAD-7))
**GAD7_Q2**<br>Not being able to stop or control worrying?	Coded Fields:<br>1. Not at all<br>2. Several days<br>3. More than half the days<br>4. Nearly every day	**Source:** iTech specific (GAD-2 Screener Q2/ GAD-7))
*Ask if **GAD7_Q1 + GAD7_Q2 ≥ 3***<br><br>**GAD7_Q3**<br>Worrying too much about different things?	Coded Fields:<br>1. Not at all<br>2. Several days<br>3. More than half the days<br>4. Nearly every day	**Source:** iTech specific (GAD-7)
*Ask if **GAD7_Q1 + GAD7_Q2 ≥ 3***<br><br>**GAD7_Q4**<br>Trouble relaxing?	Coded Fields:<br>1. Not at all<br>2. Several days<br>3. More than half the days<br>4. Nearly every day	**Source:** iTech specific (GAD-7)
*Ask if **GAD7_Q1 + GAD7_Q2 ≥ 3***<br><br>**GAD7_Q5**<br>Being so restless that it is hard to sit still?	Coded Fields:<br>1. Not at all<br>2. Several days<br>3. More than half the days<br>4. Nearly every day	**Source:** iTech specific (GAD-7)
*Ask if **GAD7_Q1 + GAD7_Q2 ≥ 3***<br><br>**GAD7_Q6**<br>Becoming easily annoyed or irritable?	Coded Fields:<br>1. Not at all<br>2. Several days<br>3. More than half the days<br>4. Nearly every day	**Source:** iTech specific (GAD-7)

Mental Health		
**Characteristic**	**Format/Values**	**Comments**
*Ask if **GAD7_Q1 + GAD7_Q2** ≥ **3***<br><br>**GAD7_Q7**<br>Feeling afraid as if something awful might happen?	Coded Fields:<br>1. Not at all<br>2. Several days<br>3. More than half the days<br>4. Nearly every day	**Source:** iTech specific (GAD-7)

Table 8: ATN Additional Measures: Social Support and Isolation

Social Support and Isolation		
**Characteristic**	**Format/Values**	**Comments**
**EMOTIONAL SUPPORT**		
**PROMIS_EmotionSupport1**<br>I have someone who will listen to me when I need to talk.	Coded Fields:<br>1. Never<br>2. Rarely<br>3. Sometimes<br>4. Usually<br>5. Always	**Source:** iTech specific (PROMIS – Emotional Support)
**PROMIS_EmotionSupport2**<br>I have someone to confide in or talk to about myself or my problems.	Coded Fields:<br>1. Never<br>2. Rarely<br>3. Sometimes<br>4. Usually<br>5. Always	**Source:** iTech specific (PROMIS – Emotional Support)
**PROMIS_EmotionSupport3**<br>I have someone who makes me feel appreciated.	Coded Fields:<br>1. Never<br>2. Rarely<br>3. Sometimes<br>4. Usually<br>5. Always	**Source:** iTech specific (PROMIS – Emotional Support)
**PROMIS_EmotionSupport4**<br>I have someone to talk with when I have a bad day.	Coded Fields:<br>1. Never<br>2. Rarely<br>3. Sometimes<br>4. Usually<br>5. Always	**Source:** iTech specific (PROMIS – Emotional Support)
**INFORMATIONAL SUPPORT**		
**PROMIS_InformationSupport1**<br>I have someone to give me good advice about a crisis if I need it.	Coded Fields:<br>1. Never<br>2. Rarely<br>3. Sometimes<br>4. Usually<br>5. Always	**Source:** iTech specific (PROMIS – Informational Support)
**PROMIS_InformationSupport2**<br>I have someone to turn to for suggestions about how to deal with a problem.	Coded Fields:<br>1. Never<br>2. Rarely<br>3. Sometimes<br>4. Usually<br>5. Always	**Source:** iTech specific (PROMIS – Informational Support)

Social Support and Isolation		
**Characteristic**	**Format/Values**	**Comments**
**PROMIS_InformationSupport3**<br>I have someone to give me information if I need it.	Coded Fields:<br>1. Never<br>2. Rarely<br>3. Sometimes<br>4. Usually<br>5. Always	**Source:** iTech specific (PROMIS – Informational Support)
**PROMIS_InformationSupport4**<br>I get useful advice about important things in life.	Coded Fields:<br>1. Never<br>2. Rarely<br>3. Sometimes<br>4. Usually<br>5. Always	**Source:** iTech specific (PROMIS – Informational Support)
**INSTRUMENTAL SUPPORT**		
**PROMIS_InstrumentSupport1**<br>Do you have someone to help you if you are confined to bed?	Coded Fields:<br>1. Never<br>2. Rarely<br>3. Sometimes<br>4. Usually<br>5. Always	**Source:** iTech specific (PROMIS – Instrumental Support)
**PROMIS_InstrumentSupport2**<br>Do you have someone to take you to the doctor if you need it?	Coded Fields:<br>1. Never<br>2. Rarely<br>3. Sometimes<br>4. Usually<br>5. Always	**Source:** iTech specific (PROMIS – Instrumental Support)
**PROMIS_InstrumentSupport3**<br>Do you have someone to help with your daily chores if you are sick?	Coded Fields:<br>1. Never<br>2. Rarely<br>3. Sometimes<br>4. Usually<br>5. Always	**Source:** iTech specific (PROMIS – Instrumental Support)
**PROMIS_InstrumentSupport4**<br>Do you have someone to run errands if you need it?	Coded Fields:<br>1. Never<br>2. Rarely<br>3. Sometimes<br>4. Usually<br>5. Always	**Source:** iTech specific (PROMIS – Instrumental Support)
**COMPANIONSHIP**		

Social Support and Isolation		
**Characteristic**	**Format/Values**	**Comments**
**PROMIS_Companionship1**<br>Do you have someone with whom to have fun?	Coded Fields:<br>1. Never<br>2. Rarely<br>3. Sometimes<br>4. Usually<br>5. Always	**Source:** iTech specific (PROMIS – Companionship)
**PROMIS_Companionship2**<br>Do you have someone with whom to relax?	Coded Fields:<br>1. Never<br>2. Rarely<br>3. Sometimes<br>4. Usually<br>5. Always	**Source:** iTech specific (PROMIS – Companionship)
**PROMIS_Companionship3**<br>Do you have someone with whom you can do something enjoyable?	Coded Fields:<br>1. Never<br>2. Rarely<br>3. Sometimes<br>4. Usually<br>5. Always	**Source:** iTech specific (PROMIS – Companionship)
**PROMIS_Companionship4**<br>Do you find companionship when you want it?	Coded Fields:<br>1. Never<br>2. Rarely<br>3. Sometimes<br>4. Usually<br>5. Always	**Source:** iTech specific (PROMIS – Companionship)
**SOCIAL ISOLATION**		
**PROMIS_Isolation1**<br>I feel left out.	Coded Fields:<br>1. Never<br>2. Rarely<br>3. Sometimes<br>4. Usually<br>5. Always	**Source:** iTech specific (PROMIS – Social Isolation)
**PROMIS_Isolation2**<br>I feel that people barely know me.	Coded Fields:<br>1. Never<br>2. Rarely<br>3. Sometimes<br>4. Usually<br>5. Always	**Source:** iTech specific (PROMIS – Social Isolation)
**PROMIS_Isolation3**<br>I feel isolated from others.	Coded Fields:<br>1. Never<br>2. Rarely<br>3. Sometimes<br>4. Usually<br>5. Always	**Source:** iTech specific (PROMIS – Social Isolation)

Social Support and Isolation		
**Characteristic**	**Format/Values**	**Comments**
**PROMIS_Isolation4**<br>I feel that people are around me but not with me.	Coded Fields:<br>1. Never<br>2. Rarely<br>3. Sometimes<br>4. Usually<br>5. Always	**Source:** iTech specific (PROMIS – Social Isolation)

Table 9: ATN Additional Measures: Motivational Pre-Exposure Prophylaxis (PrEP) Cascade

Motivational PrEP Cascade		
**Characteristic**	**Format/Values**	**Comments**
**Instructions/Introduction to Motivational PrEP Cascade:**<br>PrEP (pre-exposure prophylaxis) is a way to prevent HIV infection. PrEP involves HIV-negative individuals taking anti-HIV medications once a day, every day to reduce the chance of HIV infection if they were exposed to HIV. Research on PrEP has shown that it significantly protects against HIV infection and should be combined with other types of protections like condoms. PrEP is currently available with a prescription and most insurance companies will cover the costs. It is recommended that individuals on PrEP go to a medical provider every 3 months for HIV/STI testing, bloodwork, and a new 3-month prescription.<br><br>Please note that PrEP is not the same as taking HIV medications for a brief period of time (e.g., 28 days) after a high risk exposure to HIV through situations such as being stuck by a contaminated needle or having unprotected sex. PrEP is intended for regular, long-term use.		
**PrEP_Q1**<br>Do you think PrEP is right for you?	Coded Field:<br>1. Yes, PrEP is definitely right for me<br>2. Yes, I think PrEP is right for me<br>3. I'm not sure if PrEP is right for me<br>4. No, I don't think PrEP is right for me<br>5. No, PrEP is definitely not right for me	**Source:** Scale it Up – Stage2b: PrEP Contemplation
**PrEP_Q2**<br>Have you ever been prescribed PrEP (HIV pre-exposure prophylaxis)?	Coded Field:<br>1. Yes, I am on PrEP right now<br>2. Yes, I was in the past, but I'm not on PrEP anymore<br>3. No, I've never been prescribed PrEP	**Source:** Scale it Up – Stage 4b: PrEP Action (This question is included in the ATN Standard Domain: HIV Negative Cascade)
**ASK IF PrEP_Q2 = 2 (used to take)**<br><br>**PrEP_Q3**<br>Did you stop taking PrEP in the last 6 months?	Coded Field:<br>1. Yes<br>2. No	**Source:** Scale it Up
**ASK IF PrEP_Q2 = 2 AND PrEP_Q3 = 1 (used to take)**<br><br>**PrEP_Q4**<br>When did you first begin taking PrEP?	Month:<br>Year:	**Source:** Scale it Up
**ASK IF PrEP_Q2 = 2 AND PrEP_Q3 = 1 (used to take)**<br><br>**PrEP_Q5**<br>When did you stop taking PrEP?	Month:<br>Year:	**Source:** Scale it Up
**ASK IF PrEP_Q2 = 2 or 3 AND AGE ≥ 18 yrs (used to take or never taken)**<br><br>**PrEP_Q6**<br>How likely would you be to take PrEP if you could get it for free?	Coded Field:<br>1. I would definitely take it<br>2. I would probably not take it<br>3. I might take it<br>4. I would probably not take it<br>5. I would definitely not take it	**Source:** Scale it Up – Stage 2a: PrEP Contemplation

Motivational PrEP Cascade		
**Characteristic**	**Format/Values**	**Comments**
**ASK IF PrEP_Q2 = 2 or 3 AND AGE < 18 yrs (used to take or never taken)**<br><br>**PrEP_Q7**<br>How likely would you be to take PrEP if you could get it for free and without your parent's knowing?	Coded Field:<br>1. I would definitely take it<br>2. I would probably take it<br>3. I might take it<br>4. I would probably <u>not</u> take it<br>5. I would definitely <u>not</u> take it	**Source:** Scale it Up – Stage 2a: PrEP Contemplation
**ASK IF PrEP_Q2 = 2 or 3 (used to take or never taken)**<br><br>**PrEP_Q8**<br>Do you plan to start taking PrEP?	Coded Field:<br>1. Yes, I will definitely start taking PrEP<br>2. Yes, I will probably start taking PrEP<br>3. I'm not sure – I might start taking PrEP<br>4. No, I will probably <u>not</u> start taking PrEP<br>5. No, will definitely <u>not</u> start taking PrEP	**Source:** Scale it Up – Stage 3b: PrEParation
**ASK IF PrEP_Q2 = 2 or 3 AND PrEP_Q8 = 1 or 2 (used to take or never taken)**<br><br>**PrEP_Q9**<br>How soon do you plan to start taking PrEP?	Coded Field:<br>1. Within a month<br>2. Within 2-3 months<br>3. Within 4-6 months<br>4. Within 7 months to a year<br>5. More than a year from now	**Source:** Scale it Up
**ASK IF PrEP_Q2 = 2 or 3 (used to take or never taken)**<br><br>**PrEP_Q10**<br>Imagine you were interested in starting PrEP. Do you know of a medical provider that would prescribe PrEP to you?	Coded Field:<br>1. Yes, definitely<br>2. Yes, probably<br>3. No, probably <u>not</u><br>4. No, definitely <u>not</u>	**Source:** Scale it Up – Stage3a: PrEParation
**ASK IF PrEP_Q2 = 2 or 3 (used to take or never taken)**<br><br>**PrEP_Q11**<br>In the past 6 months have you talked to a medical provider about starting PrEP?	Coded Field:<br>1. Yes, and we both thought it was right for me and I should start PrEP<br>2. Yes, and we both thought it might be right for me but to wait before beginning PrEP<br>3. Yes, and we both thought it was not right for me<br>4. Yes, and the provider was not comfortable prescribing PrEP for me<br>5. Yes, and the provider thought it was right for me but I chose not to do it<br>6. No, I have never spoken to a provider about starting PrEP	**Source:** Scale it Up – Stage 4a: PrEP Action

Motivational PrEP Cascade		
**Characteristic**	**Format/Values**	**Comments**
**ASK IF PrEP_Q2 = 1 (currently taking)**<br><br>**PrEP_Q12**<br>When did you first begin taking PrEP?	Month:<br>Year:	**Source:** Scale it Up (This question is included in the ATN Standard Domain for the HIV Negative Cascade)
**ASK IF PrEP_Q2 = 1 (currently taking)**<br><br>**PrEP_Q13**<br>In the past 6 months have there been any times when you stopped taking PrEP?	Coded Field:<br>1. Yes, please describe when and the reasons why: ____________________<br>2. No	**Source:** Scale it Up
**ASK IF PrEP_Q2 = 1 (currently taking)**<br><br>**PrEP_Q14**<br>In the last 30 days, on how many days did you miss at least one dose of any of your PrEP? [Drop down, 0 to 30 days]	Drop down, 0 to 30 days<br><br>0 - I have not missed any doses in the past month	**Source:** Scale it Up – Stage5a: PrEP Maintenance
**ASK IF PrEP_Q2 = 1 (currently taking)**<br><br>**PrEP_Q15**<br>How regularly do you return to the medical provider who prescribes your PrEP to receive HIV/STI testing?	Coded Field:<br>1. At least 4 times per year (every 3 months)<br>2. About 2-3 times per year (every 4-6 months)<br>3. About once per year (every 12 months)<br>4. Less often than once per year	**Source:** Scale it Up – Stage5b: PrEP Maintenance
